# Manganese systemic distribution is modulated in vivo during tumor progression and affects tumor cell migration and invasion in vitro

**DOI:** 10.1038/s41598-021-95190-5

**Published:** 2021-08-04

**Authors:** Mariana Paranhos Stelling, Mariana Alves Soares, Simone Coutinho Cardoso, Juliana Maria Motta, Joice Côrtes de Abreu, Maria Júlia Mansur Antunes, Vitória Gonçalves de Freitas, João Alfredo Moraes, Morgana Teixeira Lima Castelo-Branco, Carlos Alberto Pérez, Mauro Sérgio Gonçalves Pavão

**Affiliations:** 1 Instituto Federal de Educação, Ciência e Tecnologia do Rio de Janeiro, Rua Senador Furtado, 121, Rio de Janeiro, 20270 Brazil; 2 Instituto de Bioquímica Médica Leopoldo de Meis, Universidade Federal do Rio de Janeiro, Rua Professor Rodolpho Paulo Rocco, 255 – Hospital Universitário Clementino Fraga Filho, Rio de Janeiro, 21941 Brazil; 3 Instituto de Física, Universidade Federal do Rio de Janeiro, Avenida Athos da Silveira Ramos, 149 - Centro de Tecnologia, bloco A, Rio de Janeiro, 21941 Brazil; 4 Redox Biology Laboratory, Instituto de Ciências Biomédicas, Universidade Federal do Rio de Janeiro, Avenida Carlos Chagas Filho, 373, Rio de Janeiro, 21941 Brazil; 5 Instituto de Ciências Biomédicas and Hospital Universitário Clementino Fraga Filho, Universidade Federal do Rio de Janeiro, Rua Professor Rodolpho Paulo Rocco, 255, Rio de Janeiro, 21941 Brazil; 6 Brazilian Synchrotron Light Laboratory (LNLS), Brazilian Center for Research in Energy and Materials (CNPEM), Campinas, SP 13083 Brazil; 7 Laboratório de Bioquímica e Biologia Celular de Glicoconjugados, Hospital Universitário Clementino Fraga Filho, Rua Professor Rodolpho Paulo Rocco, 255, 4º andar, sala 4A-08, Cidade Universitária, Rio de Janeiro, Rio de Janeiro CEP 21941-913 Brazil

**Keywords:** Biochemistry, Cancer, Cell biology, Molecular biology

## Abstract

Metastatic disease remains the leading cause of death in cancer and understanding the mechanisms involved in tumor progression continues to be challenging. This work investigates the role of manganese in tumor progression in an in vivo model of tumor growth. Our data revealed that manganese accumulates within primary tumors and secondary organs as manganese-rich niches. Consequences of such phenomenon were investigated, and we verified that short-term changes in manganese alter cell surface molecules syndecan-1 and β1-integrin, enhance collective cell migration and invasive behavior. Long-term increased levels of manganese do not affect cell growth and viability but enhance cell migration. We also observed that manganese is secreted from tumor cells in extracellular vesicles, rather than in soluble form. Finally, we describe exogenous glycosaminoglycans that counteract manganese effects on tumor cell behavior. In conclusion, our analyses describe manganese as a central element in tumor progression by accumulating in Mn-rich niches in vivo, as well as in vitro, affecting migration and extracellular vesicle secretion in vitro. Manganese accumulation in specific regions of the organism may not be a common ground for all cancers, nevertheless, it represents a new aspect of tumor progression that deserves special attention.

## Introduction

Cell migration is a relevant aspect of cancer^1^, it participates in tumor progression since the very early steps of tumor microenvironment formation^2^ with the recruitment of local cells and arrival of distant cells. Integrins are central molecules in migration connecting the extracellular matrix with the cytoskeleton. They mediate tumor microenvironment formation and arrival of inflammatory and metastatic cells into the healthy microenvironment^3^. Therefore, understanding the mechanisms of integrin activation is essential for the study of tumor progression. Integrins are modulated by divalent cations that bind to distinct sites and regulate its function^4^. Divalent cations are important for stabilizing integrin structure and modulating integrin-binding to its ligand, either enhancing or suppressing said binding^5^. Specific concentrations of Ca^2+^ usually present inhibitory effect, while Mn^2+^ enhances integrin-ligand binding by shifting integrins into high-affinity conformation^5^. The role of metals in cancer progression remains to be further investigated.

Primary tumors secrete molecules that promote microenvironment modulation, producing suitable niches for metastasis^6, 7^. Recent findings have shown that extracellular vesicles (EVs)^8^ released by primary tumors present a relevant role in tumorigenesis and pre-metastatic niche formation^6, 9^, a tumoral modulation of distant sites^10, 11^. Melanoma-derived EVs, for instance, were designated relevant in the promotion of metastatic niche formation^12^. Another important aspect of premetastatic niche formation is the array of cell surface and extracellular matrix molecules involved in tumor cell adhesion and migration. Integrins, cadherins, fibronectins and laminins are central molecules in this process, however, the cytoskeleton and cytoskeleton-associated proteins are also essential for directional activity towards a chemokine source^13, 14^.

In this work we investigate tumor progression from an underexplored point of view: metals modulation by the primary tumor and its systemic influence. Our findings highlight manganese as a relevant character in tumor progression, participating in enhanced tumor cell migration and forming manganese-rich niches in primary tumors and distant organs.

## Results

### Tumor-bearing mice present altered manganese systemic distribution

Multi-elemental quantification and mapping of tissue sections from healthy and tumor-bearing mice were achieved by X-ray fluorescence (XRF). Animals were analyzed from 0 to 5 weeks of tumor progression, a time frame that allows observation of early to late stages of malignancy. We observed that average Mn concentration (ppm) is not affected by tumor presence in lungs and livers, (*SI Appendix*, Fig. S1, and Table S1), however, primary tumors present increased Mn average concentration from 3 to 5 weeks of tumor progression (*SI Appendix*, Fig. S1), confirming that this structure is accumulating Mn in a time-dependent manner.

**Figure 1:**
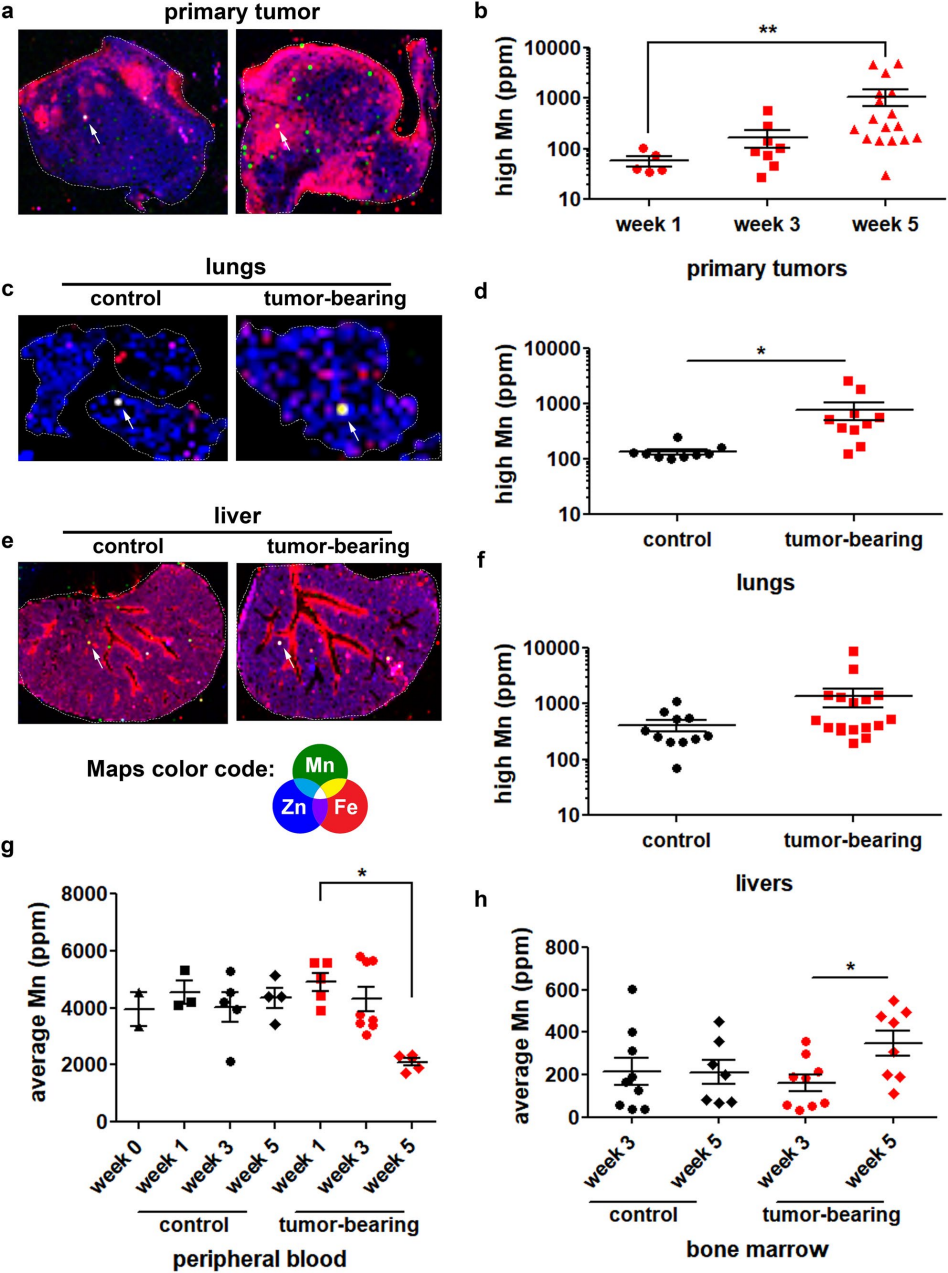
Manganese distribution is affected during tumor progression. Control and tumor-bearing mice were analyzed regarding elemental tissue content and distribution by X-ray fluorescence. Elemental distribution maps from (**a**) primary tumors—examples from two tumor-bearing mice; (**c**) lungs—from one control and one tumor-bearing mice; and (**e**) livers—from one control and one tumor-bearing mice were built from the acquired spectra; map color code: green (Mn—manganese), red (Fe—iron) and blue (Zn—zinc). Maps’ dimensions for primary tumor: 7.6 mm × 6.0 mm (left panel) and 6.9 mm × 7.7 mm (right panel); lungs: 8.6 mm × 6.2 mm (control) and 3.2 mm × 4.5 mm (tumor-bearing); livers: 11.0 mm × 14.0 mm (control) and 9.3 mm × 13.7 mm (tumor-bearing). White arrows indicate regions where manganese was found to be in highest concentration (high-Mn niches). Elemental concentration of manganese from these regions were plotted for all (**b**) primary tumors, (**d**) lungs, (**f**) livers, (**g**) peripheral blood and (**h**) bone marrow. Primary tumors were analyzed from week 1 to week 5, lungs and livers were analyzed at week 5 of tumor progression; peripheral blood was analyzed from week 0 to week 5 of tumor progression and bone marrows were analyzed at weeks 3 and 5 of tumor progression. Control mice samples are represented in black symbols, tumor-bearing mice samples are represented in red symbols. Units are expressed in concentration as ppm (parts per million). *p < 0.05; **p < 0.01, Kruskal–Wallis test and Dunn’s multiple comparison post-test. Primary tumors (week 1) N = 5, (week 3) N = 9, (week 5) N = 17; Lungs (control) N = 9, (tumor-bearing) N = 10; Livers (control) N = 11, (tumor-bearing) N = 17; Peripheral blood (week 0) N = 3, (week 1/control) N = 3, (week 1/tumor-bearing) N = 5, (week 3/control) N = 5, (week 3/tumor-bearing) N = 8, (week 5/control) N = 4, (week 5/tumor-bearing) N = 5; Bone marrow (week 3/control) N = 9, (week 3/tumor-bearing) N = 9, (week 5/control) N = 7, (week 5/tumor-bearing) N = 8.

Next, we investigated if Mn accumulation occurs heterogeneously within primary tumors. We screened samples for regions of highest Mn concentration (Fig. 1). We observed that primary tumors present regions rich in Mn (Fig. 1a), stated as ‘Mn-rich niches’, which present increased Mn concentration in a time-dependent manner (Fig. 1b and *SI Appendix*, Table S2). Interestingly, when analyzing distant organs, we noted that they also present Mn-rich niches (Fig. 1c, e) in both control and tumor-bearing mice. Lungs of tumor-bearing animals presented significantly increased Mn concentration within Mn-rich niches (Fig. 1d and *SI Appendix*, Table S2). Liver sections from control and tumor-bearing mice also presented regions of Mn accumulation (Fig. 1e) however, statistical significance was not reached (Fig. 1f—p=0.16, unpaired Student’s T-test—and *SI Appendix*, Table S2).

A time-dependent detailed multi-elemental analysis of these Mn-rich niches was performed, and we found that niches found within primary tumors present other altered elements, while lungs and livers present only altered Mn. Primary tumor Mn-rich niches presented a decrease in P from week 1 to 5, while the elements K, Fe and Cu presented an increase, and were characterized to be positively correlated with Mn, while P was negatively correlated (Table 1).

**Table 1:** Analysis of elemental composition within Mn-rich niches.

	Primary tumors	Lungs	Livers
Comparison parameters	Weeks 1–5	Control and tumor-bearing groups at week 5	Control and tumor-bearing groups at weeks 1, 3 and 5
P	Decreased, p=0.028*	Unaltered	Unaltered
S	Unaltered	Unaltered	Unaltered
K	Increased, p=0.004*	Unaltered	Unaltered
Ca	Unaltered	Unaltered	Unaltered
Fe	Increased, p=0.001*	Unaltered	Unaltered
Cu	Increased, p=0.006*	Unaltered	Unaltered
Zn	Unaltered	Unaltered	Unaltered

^*^Kruskal–Wallis test.

Peripheral blood and bone marrow were also analyzed, revealing that peripheral blood remained stable in control mice along the 5 weeks of the experiment, while tumor-bearing mice showed a decrease in Mn content at week 5 (Fig. 1g and *SI Appendix*, Table S1). At this advanced tumoral stage, we also observed decreased blood iron (Fe) levels (2.06 ratio control/tumor-bearing mice at week 5), suggesting that decreased Mn levels may not be due to a direct regulation by the tumor, rather than indirectly affected processes such as Mn/Fe absorption and transport. Bone marrow, on the other hand, presented a discrete, however significant, increase in Mn content from week 3 to week 5 in tumor-bearing mice (Fig. 1h and *SI Appendix*, Table S1).

Observed alterations indicate that the presence of a primary tumor induced by the inoculation of LLC cells into C57BL/6 mice systemically affects Mn distribution within the organism in a time-dependent manner. Mn-rich niches are found within primary tumor-specific regions, lungs and bone marrow.

### Manganese affects tumor cell migration in an in vitro model of tumor progression

To understand which possible tumor-related mechanisms were affected by Mn, we decided to analyze LLC cell behavior in vitro. Analyses were performed as close as possible to in vivo conditions, culturing and testing LLC cells in full medium conditions and observing the effect of Mn-enriched medium on cell growth and behavior.

**Figure 2:**
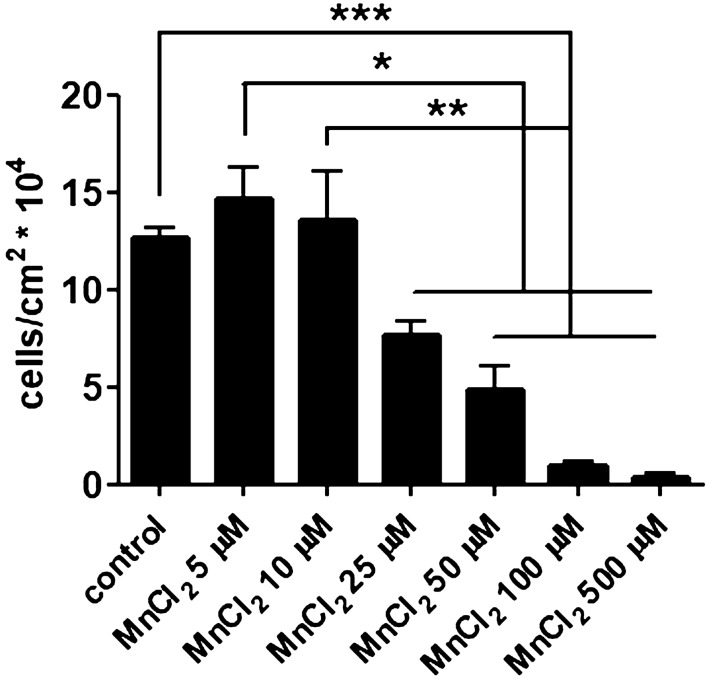
Manganese affects tumor cell survival. LLC cell survival was evaluated after 24 h of incubation with different MnCl_2_ concentrations. Cell number represents adhered live cells only. *p < 0.05; **p < 0.01; ***p<0.001, two-way ANOVA and Bonferroni’s multiple comparison post-test. N = 3.

First, LLC cells were exposed to increasing MnCl_2_ concentrations and cell growth was evaluated after 24 h of incubation (Fig. 2). We observed that LLC cell growth was not affected by added concentrations of MnCl_2_ up to 10 µM. Therefore, we supplemented 5 µM of MnCl_2_ to complete cell medium in the following experiments.

**Figure 3:**
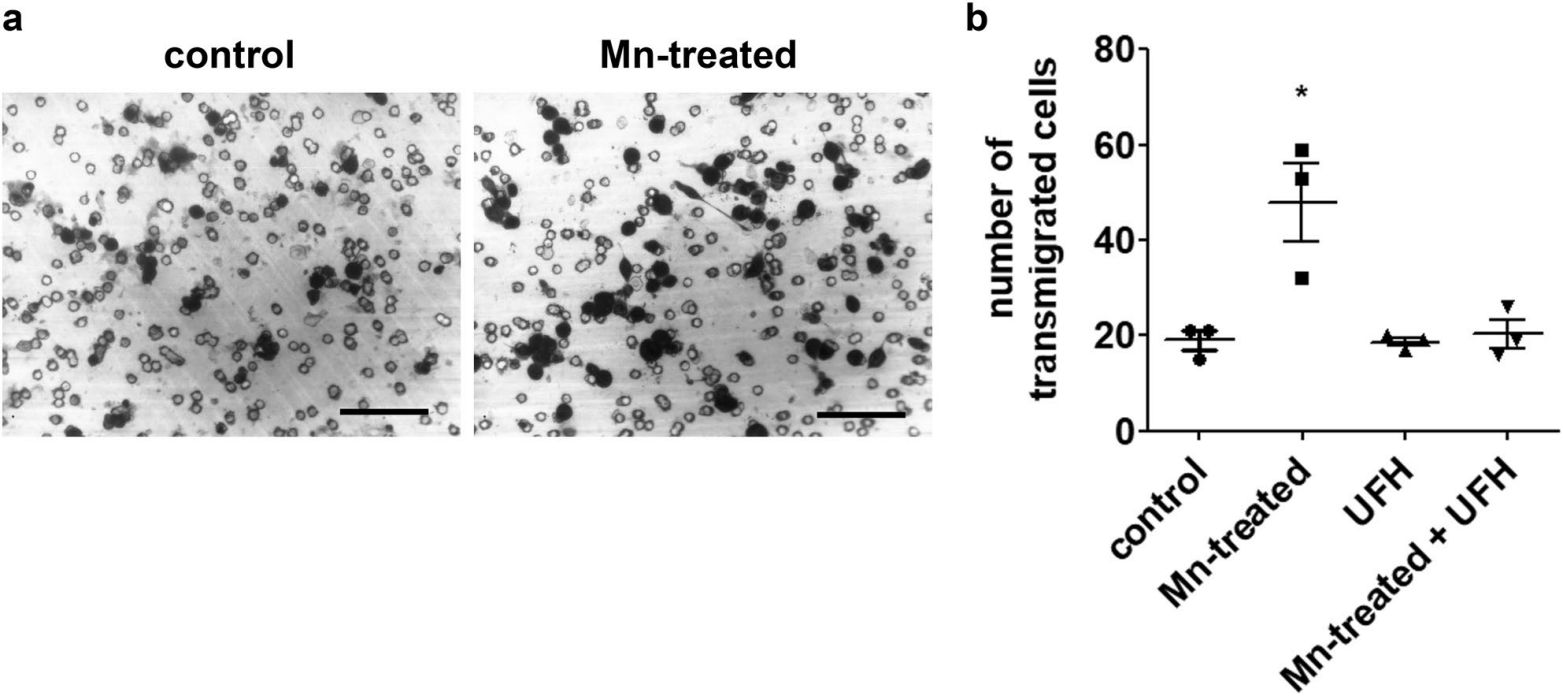
Manganese promotes tumor cell migration in vitro and heparin counteracts its effects at a non-anticoagulant concentration. LLC cells migration was evaluated in matrigel-covered transwell chambers. Transmigration was analyzed after 3 h of incubation in control and Mn-treated (MnCl_2_ 5 µM) conditions. Transwell inserts were (**a**) stained and imaged for (**b**) cell quantification. N = 3. UFH (bovine unfractionated heparin—0.1 ng/mL). Scale bars 50 µm. *p < 0.05, one-way ANOVA and Bonferroni’s multiple comparison post-test.

Next, we decided to investigate if Mn, described as an integrin activator, would affect cell migration. LLC cells were submitted to an in vitro transwell assay (Fig. 3a) and were able to transmigrate through the matrigel and pore layers. Cells exposed to Mn during the assay were more efficient in transmigrating to the bottom chamber (Fig. 3b).

To further understand Mn migration promoting effect, we decided to treat LLC cells with heparin. Heparin is a sulfated glycosaminoglycan closely related to heparan sulfate, a well-known integrin cofactor^15^. Cells were co-exposed to Mn and unfractionated heparin (UFH) at 0.1 ng/mL, a concentration with no anticoagulant activity, and transmigration was quantified (Fig. 3b). We were able to observe that UFH was capable of blocking MnCl_2_ effect on cell invasion.

**Figure 4:**
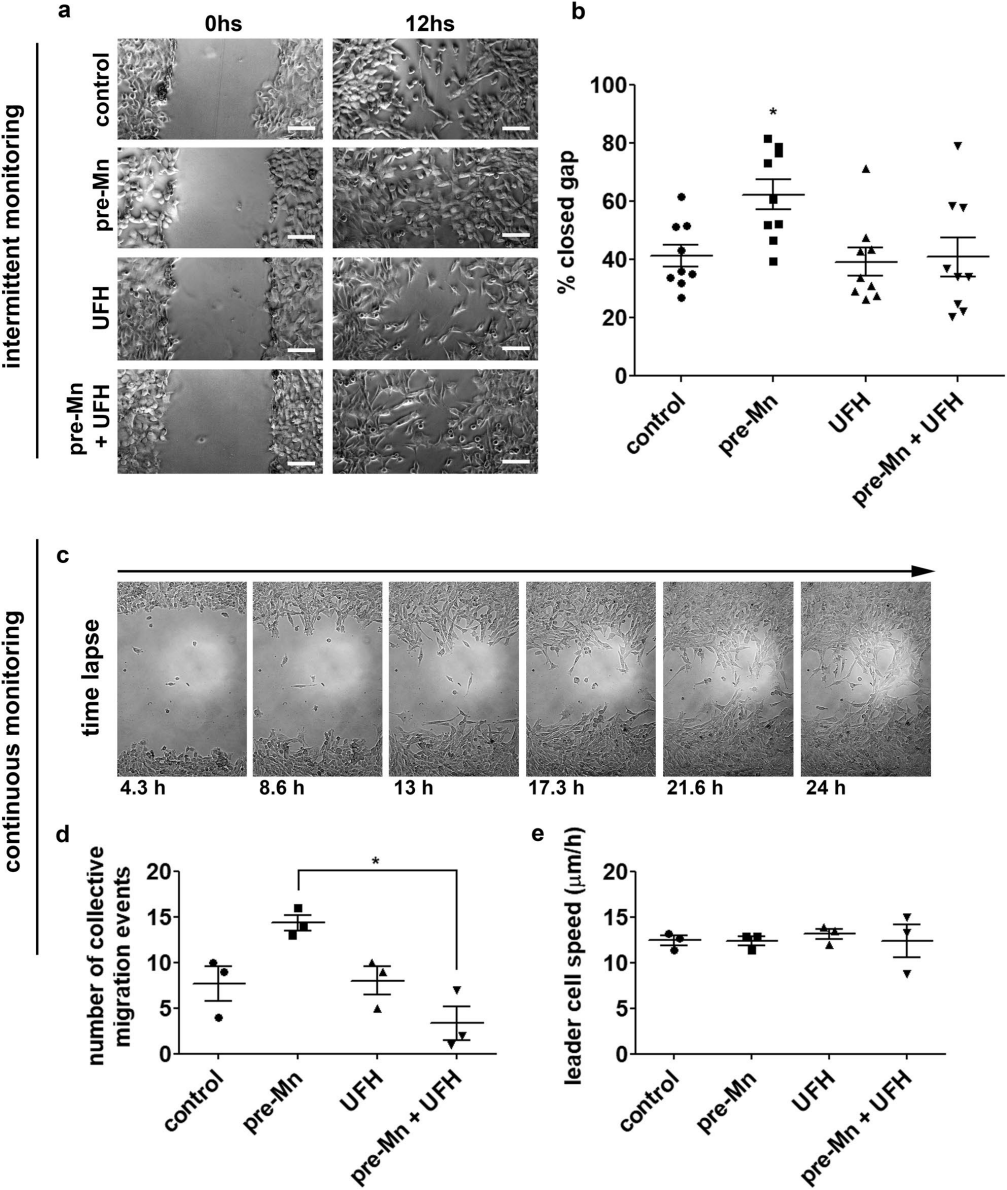
Manganese modulates tumor cell migration pattern. LLC cells migration pattern was evaluated in wound healing assays by intermittent (**a**, **b**) and continuous (**c**–**e**) monitoring. Cells were (**a**) imaged and (**b**) total migrated distance was quantified. N = 9. (**c**) Time lapse images—example from a Mn-pretreated cells migration video—were also acquired and compilated into videos for (**d**) collective migration quantification. (**e**) Single-cell speed was calculated from videos. N = 3. UFH (bovine unfractionated heparin—0.1 ng/mL); pre-Mn (MnCl_2_ 5 µM 1 h pretreatment prior to migration). Scale bars 50 µm. *p < 0.05, one-way ANOVA test and Bonferroni’s multiple comparison post-test.

Next, we performed wound healing assays (Fig. 4). Cells were pretreated with MnCl_2_ for 1 h, then this Mn-supplemented medium was exchanged to regular medium by rinsing. Cells were monitored for the migrated distance at 0 h and 12 h (intermittent monitoring—Fig. 4a). MnCl_2_ treatment induced LLC cells to migrate a greater distance compared to control cells (Fig. 4b). We have also performed time-lapse acquisitions (continuous monitoring) from which videos were created and analyzed for single-cell speed and behavior (Fig. 4c–e and *SI Appendix*, movies S1–S4). Interestingly, we observed that Mn-pretreated cells presented an increased number of collective migration events compared to control or UFH-treated cells (Fig. 4d). Leader cells were analyzed regarding actual speed (Fig. 4e) and we did not detect significant differences between tested conditions (control cells—26.4 ± 0.7 µm/h; Mn pre-treated cells—28 ± 1 µm/h; UFH-treated cells—26 ± 1 µm/h, Mn pre-treated/UFH-treated cells—26 ± 2 µm/h). The fact that Mn-pretreated cells present pronounced collective migration behavior without alterations in cell speed reveals that Mn enhances cell invasiveness by stimulating collective migration rather than affecting cell speed.

Other tumor cell lines were evaluated regarding their migration potential in wound healing assays after brief exposure to MnCl_2_-supplemented medium (*SI Appendix*, S2a–c). Data revealed that HeLa cells present similar behavior compared to LLC cells. On the other hand, B16 and MDA-MB-231 cell lines do not respond significantly to Mn, an indication that underlining mechanisms regulate cell migration.

We have also tested tumor cell behavior in the presence of other divalent metals known to affect integrin function, magnesium (Mg) and zinc (Zn). We could observe that MgCl_2_ does not affect tumor cell survival or migration (*SI Appendix*, S3a, b), while ZnCl_2_ is cytotoxic at 500 µM, but does not affect cell migration (*SI Appendix*, S3c, d) under tested conditions.

**Figure 5:**
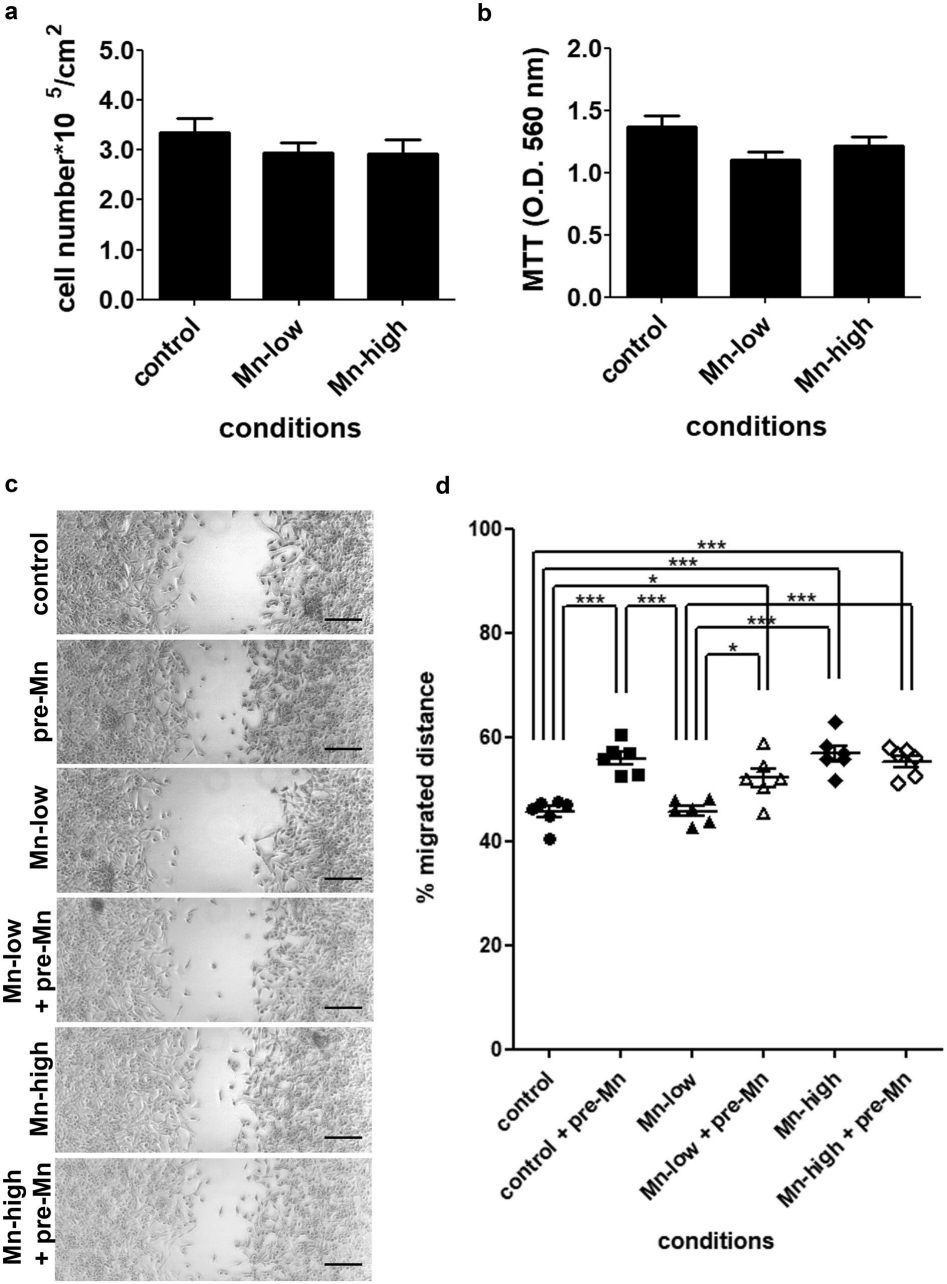
Tumor cells cultured in manganese-low and manganese-high conditions present different migration patterns. LLC cells were cultured in Mn-low and Mn-high conditions for 48 h and (**a**) cell growth was evaluated by counting live, adhered cells using trypan blue. (**b**) Cell viability was evaluated by the MTT assay. LLC cell migration pattern was evaluated in wound healing assays by intermittent monitoring at 0 h and 12 h. Cells were (**c**) imaged and migrated distance was (**d**) quantified. Mn-low (standard high glucose DMEM + Mn-low FBS); Mn-high (standard high glucose DMEM + Mn-high FBS). Scale bars 200 µm. Cell growth assay N = 4; MTT assay N = 6; wound healing assay N = 6. *p<0.05; ***p<0.0001, one-way ANOVA test and Bonferroni’s multiple comparison post-test.

To further investigate Mn influence on LLC cell migration, modified culture media were prepared with fetal bovine serum (FBS) reduced to 0.7× (Mn-low) or increased in 9.5× (Mn-high) standard Mn concentration (*SI Appendix*, Table S3). LLC cells growth and viability were assessed in Mn-low and Mn-high media for 48 h. After this period, viable LLC cells were quantified (Fig. 5a) and cell viability was assessed by MTT assay (Fig. 5b). Mn-low and Mn-high media do not affect LLC cell growth and viability in 48 h of culture. Next, LLC cells were cultured in Mn-low or Mn-high media for 24 h and migration was assessed by wound healing assays (Fig. 5c). Interestingly, migration (Fig. 5d) in Mn-low conditions is similar to control, however, when pretreated with Mn (pre-Mn), migration is slightly, but significantly affected (pre-Mn 56 ± 1.22% vs. Mn-low + pre-Mn 52.2 ± 1.82%; average ± s.e.m.). On the other hand, LLC cells cultured in Mn-high conditions migrate more, even in the absence of a Mn pretreatment (control 45.65 ± 1.11% vs. Mn-high 56.93 ± 1.5% vs Mn-high + pre-Mn 55.4 ± 1.12%; average ± s.e.m.). These data confirm that LLC cell migration is, to some extent, directly affected by Mn availability.

### Manganese is preferentially secreted from tumor cells in extracellular vesicles

**Figure 6:**
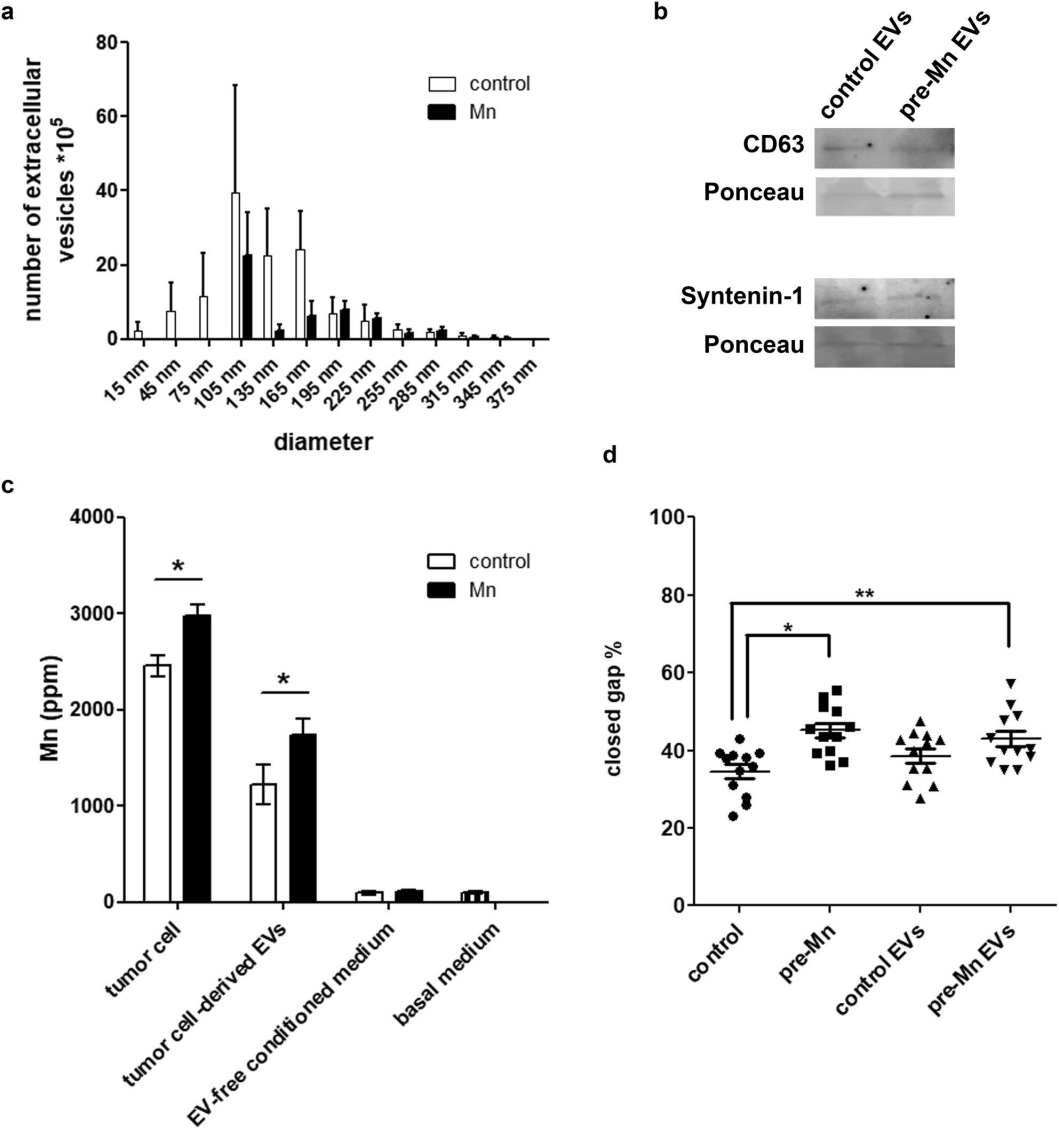
Tumor cell-derived extracellular vesicles are enriched in manganese and affect tumor cell migration. LLC cells-derived extracellular vesicles were concentrated by ultracentrifugation. (**a**) EVs quantification and size determination. N = 6. (**b**) CD63 and syntenin-1 detection by Western blotting. Ponceau was used as total protein control. (**c**) Tumor cells, EV-enriched fraction, EV-free conditioned medium and basal medium were analyzed by X-Ray Fluorescence. N = 6. Units are expressed in concentration as ppm (parts per million). Control cells—white bars; Mn-pretreated cells (Mn) – black bars. *p < 0.05, Student’s T test. (**d**) Wound healing assays of LLC cells incubated with extracellular vesicle-enriched medium from control and Mn-pretreated LLC cells. N = 12. *p < 0.05; **p < 0.01, one-way ANOVA and Bonferroni's Multiple Comparison Test.

In vivo data revealed the presence of Mn-rich niches within primary tumors and distant organs, however, only niches found within primary tumors presented alterations in other elements alongside Mn, while niches found within lungs and livers only presented alterations regarding Mn content (Table 1). Therefore, we sought to further understand the formation of local (primary tumor) and distant Mn-rich niches by investigating Mn mechanisms of distribution, such as extracellular vesicles (EVs) or secreted in soluble form. First, we incubated LLC cells with MnCl_2_-supplemented medium and after 4h of conditioning, cells were collected and analyzed for Mn content. A similar protocol was performed; however, medium conditioning took place for 24 h in basal FBS-free medium, which was submitted to an EV isolation protocol. Both control and Mn-pre-exposed cells secreted EVs in a large size-range, compatible with the secretion of mostly exosomes, but also microvesicles (Fig. 6a). In order to confirm EVs identity, an immunoblotting assay was performed for the detection of EV markers CD63 and syntenin-1 (Fig. 6b). Next, all samples were prepared for Mn quantification by XRF (Fig. 6c), whereas LLC cells exposed to Mn-pretreatment presented increased levels of retained Mn.

Interestingly, other tumor cell lines were tested (*SI Appendix*, S2d-f) and we observed that similar to LLC cells, HeLa cells retain Mn after brief exposure and show increased migration (*SI Appendix*, S2a and d), whereas B16 cells retain Mn after brief exposure, but do not respond with accentuated migration (*SI Appendix*, S2b and e). Finally, MDA-MB-231 cells do not retain Mn and do not migrate more compared to control cells *(SI Appendix*, S2c, f).

Next, we were able to verify that Mn-pretreated LLC cells secreted EVs with elevated Mn concentration (1.42 ratio Mn-pretreated/control cells), whereas the remaining EV-free medium did not present detectable differences between conditions (41 ± 8; 38 ± 2; 43 ± 8 nM Mn for basal medium, control conditioned, and pre-Mn treated conditioned medium, respectively, mean ± s.e.m.—Fig. 6c). Additionally, all the other detected elements (P, S, K, Ca, Fe, Cu and Zn) did not present differences in concentration between control EVs and pre-Mn EVs.

Finally, to evaluate the contribution of Mn-enriched EVs to tumor cell migration, we performed wound healing assays in the presence of LLC-derived EVs. The amount of EVs used for migrations were 2.5*10^8^ ± 6.2*10^7^ for control EVs and 2.9*10^8^ ± 1.5*10^8^ for pre-Mn EVs, which corresponds to 4 times the number of vesicles LLC cells would naturally secrete in culture. We could observe that naive LLC cells exposed to EVs derived from Mn-pretreated cells migrate significantly more compared to control cells (Fig. 6d).

### Manganese and heparin affect β1-Integrin and the heparan sulfate proteoglycan syndecan-1 during tumor cell migration

To unveil possible mechanisms related to Mn effect on tumor cell migration, we analyzed the expression pattern of the migration-related molecules β1-integrin and syndecan-1, as well as F-actin, in wound healing assays. PCR analyses were performed in order to confirm the presence of ITGB1 and SDC1 mRNA in LLC cells (*SI Appendix*, S4a). Next, we analyzed syndecan-1 expression in LLC cells by flow cytometry (*SI Appendix*, S4b, c). We have followed the wound healing assay protocol and collected cells at 8 h of migration. Syndecan-1 expression did not present significant changes between conditions when analyzing total cell population.

**Figure 7:**
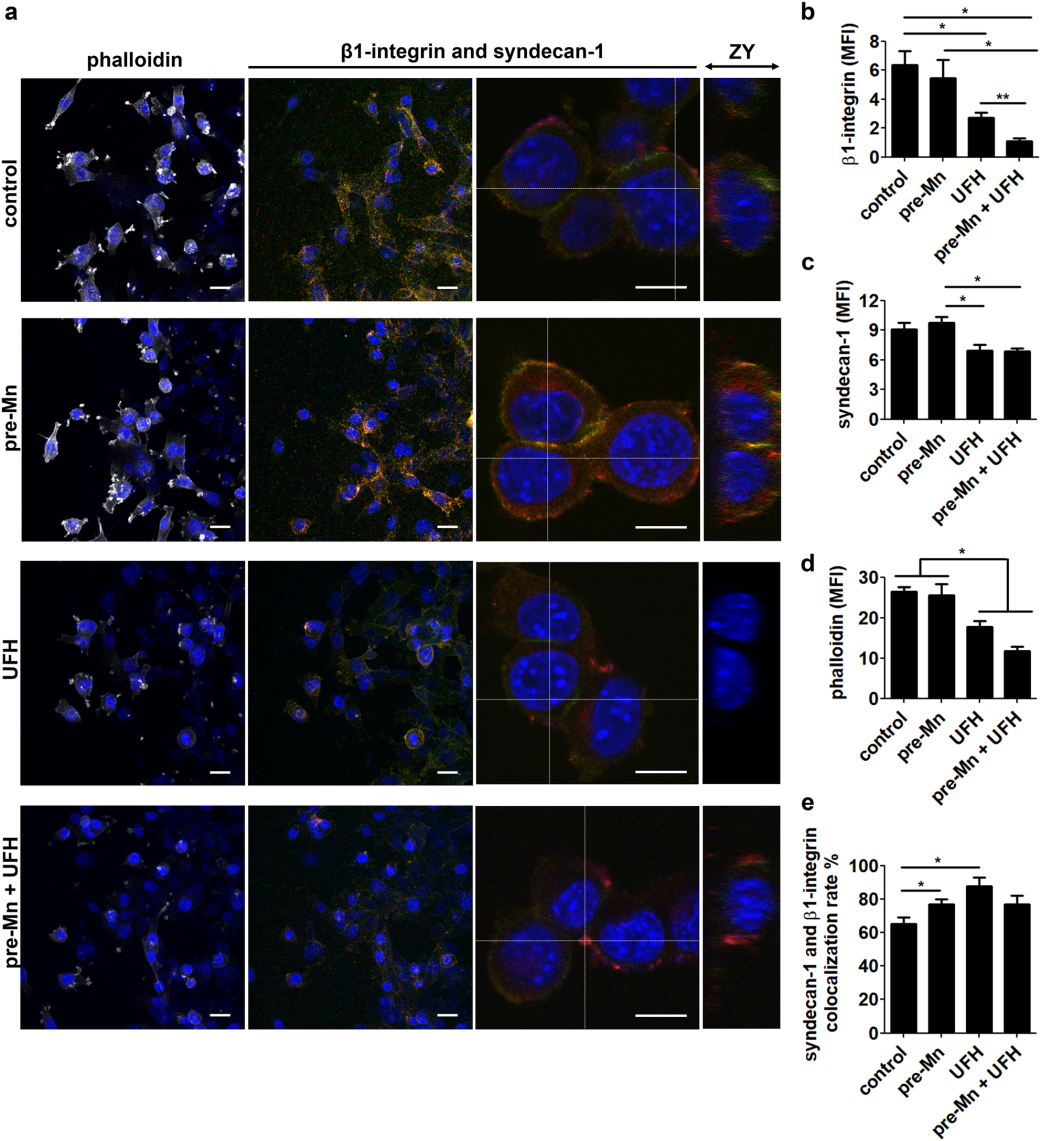
Manganese affects β1-integrin and syndecan-1 expression in tumor cells. Confocal microscopy images of (**a**) immunostainings for β1-integrin, syndecan-1 and F-actin (phalloidin). DAPI (blue), β1-integrin (red), syndecan-1 (green) and phalloidin (white). ZY axis projections were generated from higher magnification images of β1-integrin and syndecan-1 merged stainings. Scale bars are 25 µm (lower magnification) and 10 µm (higher magnification). N = 6. Images were quantified regarding fluorescence intensity for (**b**) β1-integrin, (**c**) syndecan-1 and (**d**) phalloidin-Alexa 488. (e) β1-integrin and syndecan-1 colocalization rates. MFI, mean fluorescence intensity. One-way ANOVA with Bonferroni’s post test. *p < 0.05; **p < 0.01.

Next, under similar conditions portrayed in Fig. 4a, LLC cells were let to migrate for 8 h, then cells were fixed and stained for the specific molecules (Fig. 7a). Interestingly, we observed that Mn-pretreated cells presented similar levels of β1-integrin and syndecan-1 staining compared to control cells (Fig. 7b, c), however, the colocalization rate of these molecules was significantly higher (Fig. 7e). On the other hand, heparin (UFH) treatment led to a decrease in β1-integrin and syndecan-1 staining (Fig. 7b, c) and, despite the fact that the colocalization rate in this condition is higher (Fig. 7e), we found a lower signal from these molecules in our analysis. Finally, phalloidin staining revealed that Mn does not alter F-actin, however, heparin (UFH) treatment significantly decreases F-actin staining (Fig. 7d).

Analysis of total cell population (*SI Appendix*, S4) has not shown differences between conditions; however, we were able to detect differences in β1-integrin and syndecan-1 expression near the migration edge. Higher magnification images revealed that these molecules are located on the cell surface, as well as within cells (Fig. 7a ZY projections and *SI Appendix*, S5 XZ projections), which could explain the differences in molecules’ expression found between total cell population and cells in the migration edge.

Primary tumors and livers were also analyzed for syndecan-1 expression. We were able to find high syndecan-1 cell clusters within primary tumors (N = 4) and tumor-bearing livers (N = 6), while analyzed control livers (N = 4) did not present similar clusters (*SI Appendix*, S6a–h). Additionally, a side-by-side comparison was made for sequential sections of a tumor-bearing mouse liver immunostained for syndecan-1 and irradiated for multi-elemental analyses. These twin images show the occurrence of 5 regions of high syndecan-1 cell clusters nearby high-Mn niches, 1 region of high syndecan-1 cell cluster distant from any high-Mn niche and 1 region of high-Mn niche distant from any high syndecan-1 cell cluster (*SI Appendix*, S6i and j). These data indicate a possible relationship between high-Mn niches and syndecan-1 in our in vivo mouse model of tumor growth.

### A dermatan sulfate found in the marine invertebrate *Styela plicata* presents similar antitumoral effect in vitro compared to bovine heparin

**Figure 8:**
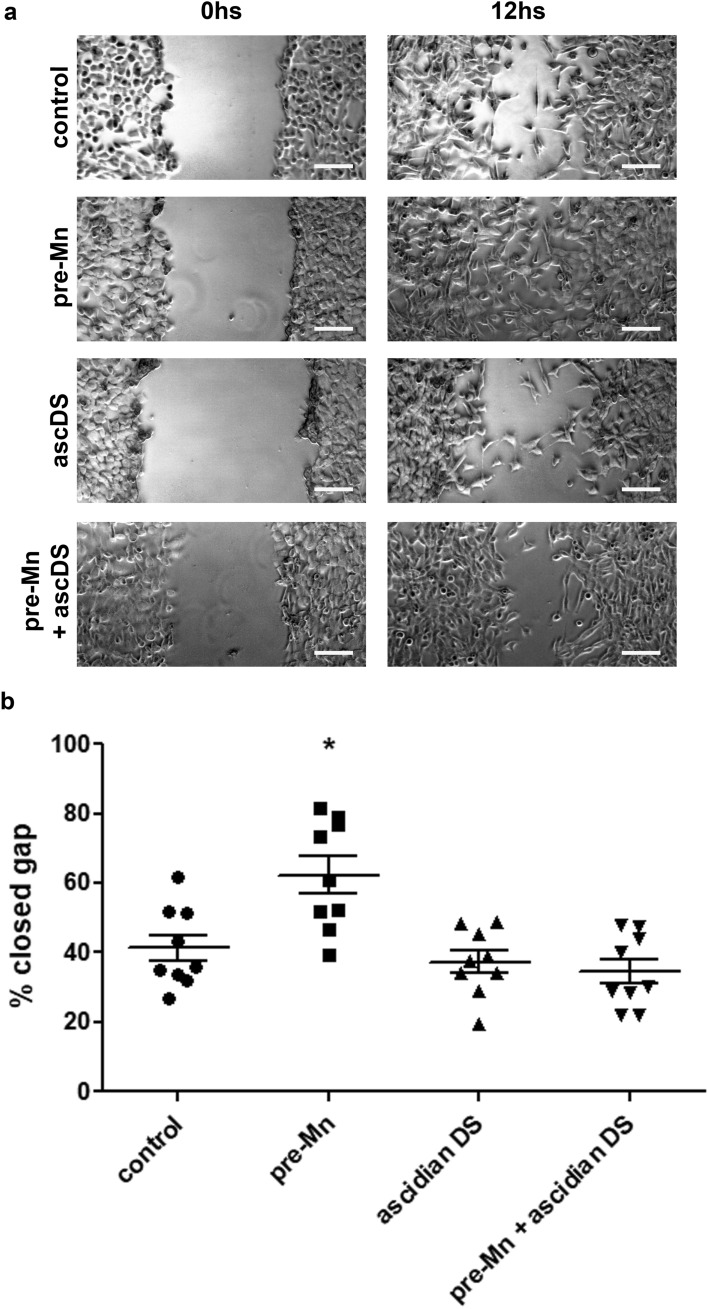
Ascidian dermatan sulfate counteracts migration-promoting effects of manganese on tumor cells. LLC cell migration was evaluated by wound healing assays after brief exposure to manganese followed by treatment with an ascidian dermatan sulfate (DS). Cells were (**a**) imaged at 0 h and 12 h and migrated distance was (**b**) quantified. N = 9. Scale bars 50 µm. *p < 0.05, one-way ANOVA test and Bonferroni’s multiple comparison post-test.

Ascidians are tunicates that produce sulfated glycosaminoglycans in their viscera. Ascidian-derived glycosaminoglycans may be considered as mammalian heparin analogs and antitumoral molecules^16^. We tested a dermatan sulfate derived from the ascidian *Styela plicata* regarding its ability to inhibit Mn-stimulated tumor cell migration in vitro. We observed that this molecule inhibits LLC cells exacerbated migration after brief Mn exposure (Fig. 8).

To investigate if UFH and the ascidian dermatan sulfate bind Mn, we performed XRF analyses on glycans that were incubated with a MnCl_2_ solution, followed by extensive dialysis. We used the structures published by Pavão et al.^17^ and Rabenstein^18^ to estimate how many Mn atoms would bind to the ascidian dermatan and to UFH. Our calculations estimated that both glycosaminoglycans efficiently retained Mn at the ratio of, approximately, one Mn atom per disaccharide, according to mass fraction estimations (*SI Appendix*, Table S4) that took into consideration the heterogeneous nature of glycosaminoglycans modifications, such as sulfate group additions. Sample contamination by free Mn was evaluated by analyzing water collected from the last dialysis day. We could observe that background levels are low and not responsible for the significant amounts detected in the glycans dialyzed solutions.

Finally, we tested if UFH would, indeed, affect Mn content and distribution in vivo. To this end, we treated tumor-bearing mice with UFH at 2 and 4 weeks of tumor development with local subcutaneous injections of UFH at a non-anticoagulant concentration. After 5 weeks of tumor development, primary tumors were collected for XRF analyses and Mn quantification and mapping. We were able to verify that both experimental groups, tumor-bearing mice (control) and UFH-treated tumor-bearing mice, presented unchanged average Mn and Mn-rich regions (*SI Appendix*, S7a; (11 ± 4)*10^2^ (untreated) vs. (28 ± 6)*10 (UFH-treated), average ± std error Mn (ppm); p = 0.23, T test); S7b; 29 ± 2 (untreated) vs. 35 ± 1 (UFH-treated), average ± std error Mn (ppm); p = 0.13, Student’s t test).

## Discussion

Trace elements, especially metals, are regulators of cellular homeostasis and present key roles on pathological processes, such as cancer^19^. They function as cofactors, electron donors and are central for bioelectrogenesis and electrophysiology. Although first reports date from many decades ago^20^, metals have recently received especial attention and, nowadays, their multiple roles are highlighted in metallomic studies^21, 22^.

The present work brings to light the relevance of manganese (Mn) modulation during tumor progression. Mn is an important cofactor for many systems, especially for integrin outside-in activation^23–26^ and Mn-dependent superoxide dismutase (SOD2) activity^27–29^. Consequently, Mn is particularly relevant in cell migration and survival, two central aspects of tumor progression and metastasis.

Interestingly, Mn has been widely studied and applied as a contrast agent for tumoral magnetic resonance imaging^30–32^. In this work we have focused our investigations on Mn distribution and accumulation in primary tumors and its effects on tumor cell behavior. We aimed to observe trace elements’ changes imposed by tumor progression and their contribution to microenvironment modulation.

In depth XRF analyses of tumor-bearing mice revealed that LLC-induced ectopic primary tumors accumulate Mn in a time-dependent manner. Accumulation occurs not only when average Mn was measured in the tissue, but much more intensely in specific regions of approximately one to four pixels (360–1440 µm^2^) in XRF imaging. Interestingly, we did not detect average Mn changes in livers and lungs. Nevertheless, when analyzing XRF elemental maps, we were able to depict Mn-rich niches, as found in primary tumors, an indication that Mn distribution is affected by tumor progression. Due to the fact that tumor-bearing mice bone marrows presented elevated average Mn levels, while peripheral blood presented a decrease at the same stage of tumor progression, we conclude that occurrence of Mn-rich niches in primary tumors and lungs, as well as elevated Mn levels in bone marrow are regulated processes strongly associated with tumor progression. Also, the fact that different tissues presented varied profiles of Mn distribution indicates that tissue Mn levels are not simply a result of Mn availability, rather, they may be the result of multiple regulation mechanisms, such as Mn transporters, Mn-binding molecules alone or associated with Mn-related ions, such as Fe, Ca and Zn.

Mn absorption and excretion are well controlled physiological processes^33^. Diet intake and gastrointestinal absorption are the main source of systemic Mn, while liver Mn processing and excretion through the gut is the main pathway for Mn losses^34^. Overall, great systemic changes in Mn are rarely observed, unless in severe intoxication cases and disease development, causing mostly neural impairment symptoms^35^. Our data revealed an interesting aspect of Mn imbalance during disease progression: occurrence of Mn-rich niches in the absence of obvious Mn systemic changes.

Mn-rich niches could be found in clusters at different locations, including near blood vessels, indicating that this element is probably originating from the circulation^36^. In addition, detailed analysis of the niches revealed that primary tumors present Mn alterations before distant organs. Also, Mn-rich niches from primary tumors present correlated alterations in P, K, Fe and Cu, while Mn-rich niches from lungs and livers only present alterations in Mn. These data indicate that Mn alterations initiate in primary tumors and may influence the occurrence of Mn-rich niches in healthy tissues.

Mn absorption, distribution and excretion are well regulated systemic processes, which points towards the formation of Mn-rich niches as a hidden process, undetectable to widely used metal detection techniques and, more importantly, to biological systemic sensing mechanisms. Our in vitro data show that even brief changes in Mn levels in culture medium allow LLC cells to significantly accumulate Mn and secrete it in extracellular vesicles (EVs), indicating a Mn route in LLC cells responsible for Mn retention, internalization, and secretion in EVs. The relevance of these data is highlighted in different works, such as by Harischandra and colleagues^37^. The authors show that Mn exposure promotes α-synuclein secretion in exosomal vesicles, which subsequently evokes proinflammatory and neurodegenerative responses. They report that, in dopaminergic neurons, Mn stimulates exosome release^37^. Additionally, exosome-associated integrins present important functions in premetastatic niche formation and metastasis. As reviewed by Hoshino et al.^38^, some works showed that exosome-associated integrin expression profiles underlie tumor cell organotropism. For instance, exosomal integrins α6β4 and α6β1 were linked to lung metastasis, while exosomal integrin αvβ5 was related to liver metastasis. Different pairs of bone marrow associated integrins, such as α4β1, were shown to be essential for hematopoietic cell migration within bone marrow^11^, therefore the detected elevated levels of Mn within tumor-bearing mice bone marrow may be due to the arrival of extracellular vesicles expressing Mn-activated integrins released by cancer cells from primary tumor. We hypothesize that LLC cells continuously retain Mn from circulation, resulting in average elevated Mn levels in primary tumors, as well as formation of Mn-rich niches through extracellular vesicle delivery. Multi-elemental analyses of Mn-pretreated cells-derived EVs revealed that Mn is the only detected element at significantly different levels compared to control EVs. This information points toward EVs as one of the likely routes of Mn distribution from the primary tumor to distant organs during tumor progression. Finally, we have analyzed different tumor cell lines and found that Mn accumulation does not occur in all cells, for instance HeLa and B16 cell lines retain Mn after brief in vitro exposure, while MDA-MB-231 cells do not. These additional data confirm the specificity of Mn retention, internalization and secretion in some types of tumors.

There are a number of pathways related with Mn distribution. The Mn transporter DMT1 is reported to be involved in tumor progression, however, most reports focus on iron, rather than Mn, as a DMT1-associated tumor-inducing agent^39–41^. ZIP (Zrt- and Irt-like protein) transporters, on the other hand, have been associated with zinc and Mn transport, and being expressed by endothelial cells^42^, are stronger candidates for Mn retention in primary tumors. Alterations in the expression levels of Mn transporters are also described to lead to altered Mn absorption. The work by Mercadante and colleagues shows that Slc30a10-deficient mice present severe Mn excess accumulation, and this phenomenon was linked to Slc30a10 deficiency in the liver and small intestines^43^. Mutated SLC39A14 has also been described to cause severe Mn accumulation in humans, leading to the early onset of parkinsonism-dystonia^44^. Our data shows that, even in a regular diet, free from Mn intoxication sources, tumor-bearing animals present a unique feature, which is the formation of Mn-rich niches. We show that these niches are formed in a time-dependent manner after Mn accumulation in the primary tumor and reach impressive levels in advanced stages of tumor progression. Further investigations of alterations in Mn transport mechanisms in tumor cells are an interesting and relevant perspective for the field.

Charge interactions are a fast and less specific pathway for Mn retention and subsequent internalization. In the present work we show that LLC cells express the heparan sulfate proteoglycan syndecan-1 and confocal analyses revealed that upon Mn treatment, syndecan-1 and β1-integrin present higher colocalization rates. Proteoglycans have been described as relevant co-receptors and their sulfated glycosaminoglycans chains offer many binding sites for Mn on the cell surface^45, 46^. Mn could be solely responsible for affecting syndecan-1 cell surface distribution pattern if proteoglycan internalization or shedding were promoted by binding to the cation. On the other hand, these processes could also be mediated by proteoglycan-mediated Mn presentation to its receptor on the cell surface. High magnification confocal analyses of syndecan-1 and β1-integrin immunostainings revealed that these molecules can be found on the cell surface, as well as intracellularly, confirming that both processes are equally possible. Finally, syndecan-1 immunostainings of primary tumors and livers from control and tumor-bearing mice indicate that this proteoglycan may be involved in Mn distribution in vitro, as well as in vivo.

Mn-rich niches in distant organs are discrete in comparison to primary tumors, nevertheless they can also be found clustered near vessels (Fig. 1e). Following the time-associated evolution of this pattern, we hypothesize that distant organs may receive Mn from primary tumors, forming Mn-rich niches later in tumor progression. Mn distribution from primary tumors can occur by secreting soluble factors or vesicles into the circulation. Our data shows that, in vitro, if briefly exposed to excess Mn, LLC cells release Mn-rich EVs. We have also detected that the remaining vesicle-free medium contains the same levels of Mn concentration as basal medium, confirming that EVs are the preferential route for Mn secretion in these cells. The release of Mn-rich EVs in the circulation would explain the observed pattern of Mn-rich niches without impacting Mn average tissue levels. Taking into consideration Mn-dependent processes of cell migration and survival, we hypothesize that occurrence of Mn-rich niches in distant organs may be a characteristic of microenvironment modulation.

Cell survival and migration is a crucial step in metastasis, and Mn is a well-established integrin activator^23, 25, 47^, an essential factor to both processes. Our data shows that LLC cells present enhanced migration when exposed to Mn-rich culture medium. Indeed, we have observed that this behavior is not repeated by incubating cells with divalent cations, magnesium and zinc, supporting the specific relevance of Mn modulation during tumor progression. We hypothesize that tumor cells able to retain Mn in vivo migrate more efficiently due to a more efficient integrin activation and collective migration behavior. Supporting evidence of this phenomenon has been described in an in vitro model of tumor cell migration, whereas MDA-MB-231 cells are exposed to MnCl_2_ and monitored for migration and proliferation activities^48^. Findings show that MnCl_2_ 5 µM mainly stimulates transmigration through type I collagen (chemotaxis assay), while transmigration through matrigel (chemoinvasion assay) is more pronounced at MnCl_2_ 100 µM. Our group has found that MDA-MB-231 cells do not retain Mn and do not present enhanced migration in the wound healing assay at MnCl_2_ 5 µM, in consonance with the results described above by Luparello (2019). Nevertheless, differences in cell behavior according to Mn concentration and ECM setup may arise from variations in Mn transport mechanisms and in the expression profile of ECM-binding cell surface molecules. LLC cells were also cultured in Mn-modified media (Mn-low and Mn-high) and we could see that cell viability was not affected by these culture conditions. Next, we verified that basal migration is not affected in Mn-low culture conditions, however, when cells are pretreated with a pulse of high Mn, Mn-low cells migrate slightly less than control cells. On the hand, LLC cells cultured in Mn-high conditions migrate similarly to pre-Mn cells (control cells + Mn pretreatment), and, interestingly, pre-Mn treatment in Mn-high cells does not further enhance migration. These data reveal that tumor cell migration is affected by both short-term and long-term Mn exposure. This model of in vitro tumor cell migration indicates that tumor cells in vivo may experience similar conditions and present enhanced migration when near or within Mn-rich niches.

Other tumor cell lines were analyzed regarding migration behavior after brief exposure to Mn-rich culture medium, interestingly, cell lines able to retain Mn may (HeLa cells) or may not (B16 cells) respond to Mn, however, among the tested cell lines the one that does not retain Mn, also does not change its migration pattern upon Mn stimulation (MDA-MB-231 cells).

Finally, we tested if exogenous sulfated polysaccharides: heparin and ascidian dermatan sulfate would act as Mn-chelating agents. These molecules are great candidates to interfere with tumor progression because they counteract Mn effects at exceptionally low concentrations, below their anticoagulant activity, in addition to being physiological metal modulators^49^. Both glycosaminoglycans bind to Mn, regulate Mn-stimulated tumor cell migration and, in the case of heparin, decrease F-actin staining during migration. However, different glycosaminoglycans may promote or inhibit tumor progression^45^. Marine-derived molecules have been studied as antitumoral agents^16^. Groult and colleagues have tested red seaweed carrageenan regarding its anti-heparanase effect on MDA-MB-231 breast cancer cells^50^. *Styela plicata* is an ascidian that produces a dermatan sulfate with structural similarities with mammalian heparin but does not share the same potent anticoagulant effect^51^.

In summary, our analyses point to a time-dependent process that promotes the formation of Mn-rich niches. These niches are prominent within primary tumors; however, they can also be observed in distant organs. Our results show changes in LLC cells migration even after a short-term exposure to Mn from invasive to highly invasive. Interestingly, β1-integrin and syndecan-1 are found colocalized after manganese short-term exposure in migrating cells. EVs also change from presenting low manganese content to higher manganese content.

In conclusion, the overall results revealed an unexplored role of Mn in tumor progression and possible mechanisms involving its effect on tumor cell motility. Mn accumulation in specific regions of the organism may not be a common ground for all cancers, nevertheless it represents a new aspect of tumor progression that deserves special attention.

## Materials and methods

### Cell lines and standard cell culture conditions

Cells were cultured under standard conditions at 37 °C and CO_2_ 5% atmosphere. Cell culture medium was composed of DMEM (Sigma-Aldrich) supplemented with fetal bovine serum (FBS) 10% (v/v) (Vitrocell) and glucose 4500 mg/L (Sigma-Aldrich). Cells were passaged with a trypsin-EDTA solution (trypsin 0.25% and EDTA 1 mM). Protease-free cell detachment was achieved by incubating cells in a Ca^2+^ and Mg^2+^-free PBS solution with EDTA 1 mM, followed by detachment with a cell scraper and further pipetting. Cell viability was assessed by counting detached cells using a Neubauer chamber in trypan blue 0.2% solution. Each quantification was performed at least three times.

Cell lines used in this work were LLC (mouse Lewis lung carcinoma, ATCC), MDA-MB-231 (human breast adenocarcinoma, ATCC), HeLa (human cervix adenocarcinoma, ATCC) and B16 (mouse melanoma, ATCC).

### Manganese cell toxicity assays

LCC cells were seeded in 24-well plates (6 × 10^4^ cells/well) and after cultures reached 80% confluence, cells were incubated for 24 h in increasing concentrations of MnCl_2_ (5, 10, 25, 50, 100 and 500 μM). Cells were harvested by enzymatic treatment and viable cells were quantified in a Neubauer chamber using trypan blue.

### Transmigration assays

Semi-confluent LLC cells were detached using the protease-free method and quantified as described above. Next, transwell inserts 8 µm pore-size (Corning) were placed on 24-well culture plates and the top chamber was incubated with 100 µL matrigel (BD Biosciences) diluted in FBS-free medium to 1 mg/mL. Inserts were incubated at room temperature for 2 h. Next, excess medium was removed by gentle aspiration and inserts were prepared for transmigration. Bottom chamber was filled with 650 µL culture medium supplemented with FBS 10%, while top chamber received 100 µL FBS-free medium with a total of 10^5^ cells. Plates were incubated in standard culture conditions for 3 h, when inserts were collected for processing. Quantification of transmigrated cells was achieved by removing inserts from culture plates, followed by gentle scraping of non-transmigrated cells from the top chamber using a cotton swab. Next, inserts were rinsed in PBS, fixed and stained in a crystal violet 0.5% (m/v) solution in methanol 20%. Stained inserts were imaged, and cell quantification was performed using ImageJ software 1.52a.

### Cell migration wound healing assays

Cells were cultured in 6-well plates until 80% confluence, then culture medium was changed to control (regular culture medium) or pre-Mn (regular culture medium with the addition of manganese chloride (MnCl_2_—Sigma-Aldrich) 5 µM. Cells were incubated in these conditions for 1 h. Next, medium (control or Mn-added) was removed, cells were rinsed and mechanically removed from the plate in a cross-shaped pattern using a sterile P1000 tip. Cells were rinsed again for removal of cell debris and incubated in control (regular culture medium) or polysaccharide-treated (regular culture medium with the addition of unfractionated bovine heparin—UFH or ascidian-derived dermatan sulfate—ascDS at 0.1 ng/mL) for 12 h. In order to evaluate cell migration and wound closure, cells were imaged, using the cross center as a reference at 0 h and 12 h. Migration was quantified using ImageJ software and expressed as the percentage of migrated distance relative to the original wound width.

An automatic microphotography system was used to monitor migrating cells under standard culture conditions and generate time lapse videos. Cells were imaged every 2 min for 24 h. Images were compiled into videos that were analyzed regarding migrating pattern (collective cell migration) and single-cell speed (ImageJ software).

### Extracellular vesicles concentration from tumor cell conditioned medium

LLC cells were cultured until 70% confluence, next, control culture medium was changed to pre-Mn culture medium and cells were incubated for 4 h. Afterwards, cells were rinsed and incubated in serum-free basal medium (high glucose DMEM) for 24 h for medium conditioning. Extracellular vesicles’ concentration was achieved by an ultracentrifugation protocol^52^ as follows: conditioned medium was centrifuged at 300*g* for 10 min, 2000*g* for 10 min, and 10,000*g* for 30 min. Pellets were discarded after each centrifugation round. Next, the remaining supernatant was ultracentrifuged (Beckman 70Ti rotor) at 100,000*g* for 70 min. The extracellular vesicle-enriched pellet was washed in PBS to eliminate contaminating proteins and centrifuged one last time at 100,000*g* for 70 min. The final pellet was resuspended in 100 µl of PBS. A Nanoparticle Tracking Analysis – NTA Zetaview - was used to monitor separation.

### Extracellular vesicles markers characterization by immunoblotting

For extracts, exosomes previously purified were lysed in 50 mM HEPES, pH 6.4, 1 mM MgCl2, 10 mM EDTA, 1% Triton X-100, 1 µg/mL DNase, 0.5 µg/mL Rnase, 1 mM PMSF, 1 mM benzamidine, 1 µg/mL leupeptin and 1 µg/mL soybean trypsin inhibitor. Total protein content in the exosomes extracts was determined by BCA method. Lysates were denatured in sample buffer (50 mM Tris·HCl, pH 6.8, 1% SDS, 5% 2-ME, 10% glycerol, and 0.001% bromophenol blue) and heated in boiling water for 3 min. Samples (10 µg total protein) were resolved by 15% SDS-PAGE and proteins transferred to polyvinylidine difluoride membranes. Molecular weight standards were run in parallel. Membranes were blocked with Tween-TBS (TBS, 0.01% Tween 20; T-TBS) containing 5% BSA and probed with primary antibody (1:500) overnight at 4 °C. Primary antibodies used in western analysis were anti-CD63 cat: 10628D and anti-Syntenin-1 cat: PA528813 (Invitrogen, Carlsbad, CA, USA). The membranes were rinsed with T-TBS and incubated for 1h at room temperature with HRP-conjugated secondary antibody (1:5000). Immunoreactive proteins were visualized by the ECL detection (GE, Chicago, IL, USA).

### Extracellular vesicles in cell migration wound healing assays

LLC cells were submitted to wound healing assays incubated with extracellular vesicles (EVs) enriched medium containing 4 times more EVs than a naturally conditioned medium. Cells received EVs from naïve LLC cells (control EVs) and from MnCl_2_ pre-exposed cells (pre-Mn EVs). In order to evaluate cell migration and wound closure, cells were imaged at 0 h and 12 h. Migration was quantified using ImageJ software and expressed as the percentage of migrated distance relative to the original wound width.

### Animal model of spontaneous metastasis

C57BL/6 male and female mice between 8 and 12 weeks of age were anesthetized and inoculated subcutaneously in the posterior dorsolateral region with 5 × 10^5^ LLC cells in 60 µL serum-free DMEM as vehicle (tumor-bearing group), whereas control animals were inoculated with vehicle only. Animals were randomly assigned to experimental groups, comprising a minimum of five specimens per experimental group in order to accurately assess variations between specimens. All experimental groups were kept under standard conditions and daily monitored for vital signs, behavior and tumor size from 0 to 5 weeks of tumor development. Animals that presented signs of possible discomfort were anesthetized with a combination of xylazine and ketamine and submitted to a cervical dislocation procedure according to the Brazilian animal experimentation guidelines. Death was confirmed by observing all of the following: absence of breathing movements, absence of pulse and absence of heartbeat. These animals were excluded from the experiments.

Tissue samples were collected in order to investigate tumor progression in a time-dependent manner. Specifically, primary tumors, liver and lungs, in addition to peripheral blood and bone marrow were harvested at weeks 0–5 and processed for analyses. Solid tissues were rinsed in saline, briefly and superficially fixed in 4% paraformaldehyde for 5 min and cryopreserved in OCT (Optimal Cutting Temperature compound – Tissue-Tek) for further sectioning. Liquid tissues were collected fresh and immediately processed for further analyses. All procedures involving animal experimentation were approved by the Federal University of Rio de Janeiro Animal Experimentation Committee (protocol number: 015/18) and were performed in accordance with the Brazilian guidelines for scientific use of animals. This work is in compliance with the ‘Animal Research: Reporting of In Vivo Experiments’ (ARRIVE) essential 10 guidelines.

### X-ray microfluorescence analyses

X-ray microfluorescence analyses were performed at the UVX Light Source at the Brazilian Synchrotron Light Source Laboratory (LNLS). Analyzed samples comprised of homogeneous liquid samples, cultured cells or tissue sections placed on Ultralene thin film (SPEX SamplePrep). Homogeneous liquids and resuspended cells were placed on Ultralene film in drops and let to air dry. Tissue samples were cryosectioned into 20 µm-thick slices and placed on Ultralene film. Cultured cell samples were quantified, scraped from the plate, placed on Ultralene film and let air dry. Samples were analyzed at D09B X-ray Fluorescence beamline at room temperature and ambient pressure according to Sartore et al.^53^.

### Immunostaining imaging

LLC cells were cultured on RS glass slides (Nunc) covered in growth factor-reduced matrigel (BD Biosciences). When 80–90% confluence was reached, cells were submitted to the migration wound healing assay for 8 h. Cells were fixed in 4% paraformaldehyde for 10 min. Next, cells were rinsed with PBS and incubated in blocking solution (2% bovine serum albumin in PBS) for 30 min, followed by incubation with Triton 0.1% for 5 min. Primary and secondary antibodies were incubated separately for 90 min each in the following order: rat anti-syndecan-1, anti-rat-Cy5, goat anti-β1-integrin, anti-goat-Cy3, phalloidin-Alexa 488. Cells were rinsed with PBS between steps. Then, slides were mounted in anti-fade mounting medium Vectashield (Vector Laboratories) with DAPI. Antibodies used for immunostaining were diluted in 0.5% albumin in PBS and are anti-CD138 (1:100 - RD Systems) and anti-β1 integrin (1:100 - Santa Cruz Biotechnology).

Images were acquired on the confocal microscope system TCS SPE (Leica) and processed using LAS X software (Leica). Cells were imaged using a 10X objective lens and a 40X oil immersion objective lens (with additional 3X zoom). Lasers used were 488, 583 and 633 nm wavelength. Images were acquired in stacks of 500 nm thickness and the number of stacks varied according to sample height.

Traditional widefield immunofluorescence images were acquired using an Axio Imager A1 microscope and Axiovision System software (both Zeiss; Oberkochen, Germany).

### Mn-low and Mn-high fetal bovine serum

LLC cells were cultured in modified cell media and evaluated for cell viability and migration pattern. Cell media were prepared with standard basal high-glucose DMEM medium and Mn-depleted (Mn-low) and Mn-supplemented (Mn-high) fetal bovine serum (FBS). Modified FBS solutions were prepared from standard FBS by treatment with activated Dowex 50Wx4 cationic exchange resin (Dow) for removal of cations^54^. Next, a mix of 80% dowex-treated FBS and 20% standard FBS was made in order to partially replenish FBS of Dowex-removed cations. Next, to recover FBS standards of cation composition, the following salts and respective concentrations were added to the mix: CaCl_2_ 3.85 µM, FeCl_2_ 21 µM, KCl 7 mM and NaCl 101.5 mM. Mn-low FBS did not receive any additional supplementation, while Mn-high received additional MnCl_2_ in order to meet average FBS Mn levels^55^. Finally, Mn-low and Mn-high FBSs were filtered using a 0.22 µm syringe filter (Millipore) in order to ensure all solutions were sterile. Mn-low and Mn-high manipulated FBS were both prepared from the same initial solution in order keep the same overall composition for both conditions, with the exception of MnCl_2_. ICP-OES analyses were performed in order to define elemental composition of modified fetal bovine sera (*SI appendix*).

### Cell survival and viability in Mn-low and Mn-high medium

LLC cells were cultured in standard conditions until experiments were performed. LLC cell survival was evaluated by seeding 2.6*10^4^ cells/cm^2^ into 6-well plates in standard cell medium. After 3 h, medium was carefully exchanged to experimental media as: control medium, Mn-low medium and Mn-high medium. Cells were cultured for 48 h and quantified in a Neubauer chamber using trypan blue.

Cell viability was evaluated using the MTT assay. LLC cells were cultured in standard and respective experimental conditions for 48 h in 96-well plates. Cell viability was assessed by incubation with tetrazolium salt (MTT, Roche) 0.5 mg/mL in cell medium for 2 h. After incubation, cell medium was discarded, and formazan crystals were dissolved in 200 µL dimethyl sulfoxide (DMSO, Sigma-Aldrich) per well. Absorbance at 560 nm was acquired using SpectraMax Plus microplate reader (Molecular Devices) and analyzed using Softmax Pro software (Molecular Devices).

### Cell migration wound healing assays in Mn-low and Mn-high medium

LLC cell migration in Mn-low and Mn-high conditions was evaluated following the same protocol described for cell migration wound healing assay. Cells were seeded onto 6-well plates in standard culture conditions and, after 24 h of incubation, medium was exchanged to control, Mn-low and Mn-high medium accordingly. After 12 h of incubation in the modified media, cells were briefly exposed to a Mn pulse by incubation in their respective medium with added MnCl_2_ 5 µM. After this brief exposure, cells were rinsed and the scratch was done. Cells were imaged at 0 h and 12 h of migration in their respective modified medium and were named: control, pre-Mn, Mn-low (no Mn pulse before migration), Mn-low + pre-Mn (Mn pulse before migration), Mn-high and Mn-high + pre-Mn, respectively.

### Ascidian dermatan sulfate isolation from *Styela plicata*

*Styela plicata* specimens were collected at Praia da Urca, Rio de Janeiro, Brazil. Ascidian dermatan sulfate (ascDS) was isolated from *Styela plicata* viscera by proteolytic digestion followed by anion exchange chromatography, as previously described^17^. Collection of ascidian specimens was authorized by the Brazilian regulatory agency ICMBio, under the protocol number 66457-1 (SISBIO).

### Sulfated glycosaminoglycans and manganese interaction assays

Unfractionated bovine heparin (UFH) and ascidian dermatan sulfate (ascDS) were tested for manganese binding. A total mass of 1 mg of each polysaccharide was incubated in MnCl_2_ 1 M for 24 h at room temperature, followed by 5 days of dialysis. X-ray fluorescence was used to assess polysaccharide-bound manganese. Water from the last dialysis day (control water) was used as a control for residual free manganese.

### Statistical analyses

Statistical analyses were performed using GraphPad Prism 5 for Windows, version 5.03 (2009). Kruskal–Wallis test with Dunn’s post test was applied to all in vivo experiments, while one-way ANOVA with Bonferroni’s post test was applied to in vitro experiments where applicable, otherwise, Student’s T test was applied for comparisons when there were only two experimental groups to be analyzed.

## Supplementary information

Supplementary Information 1.

Supplementary Video S1.

Supplementary Video S2.

Supplementary Video S3.

Supplementary Video S4.
