# Supplementary material for: Manganese systemic distribution is modulated in vivo during tumor progression and affects tumor cell migration and invasion in vitro
Source: Sci Rep. 2021 Aug 4;11:15833. doi: 10.1038/s41598-021-95190-5 (PMC8338990; doi:10.1038/s41598-021-95190-5)
Supplement: Supplementary file 1 — Supplementary Information 1. [file 41598_2021_95190_MOESM1_ESM.pdf]

Supplementary Information for

Manganese systemic distribution is modulated *in vivo* during tumor progression and affects tumor cell migration and invasion *in vitro*

Mariana Paranhos Stelling[#1,2], Mariana Alves Soares[#2], Simone Coutinho Cardoso[#3], Juliana Maria Motta[2], Joice Côrtes de Abreu[2], Maria Júlia Mansur Antunes[2], Vitória Gonçalves de Freitas[1,2], João Alfredo Moraes[4], Morgana Teixeira Lima Castelo-Branco[5], Carlos Alberto Pérez[6], Mauro Sérgio Gonçalves Pavão[2]

[#]These authors contributed equally to this work.

Mauro Sérgio Gonçalves Pavão
Email: mpavao@hucff.ufrj.br

This file includes:

Supplementary Methods
Supplementary Figs. S1 to S7
Supplementary Tables S1 to S4
Captions for supplementary movies S1 to S4

Other supplementary materials for this manuscript include the following:

Movies S1 to S4

Supplementary Methods

Wound healing assays for tumor cell lines HeLa, B16 and MDA-MB-231

The different cell lines HeLa, B16F1 and MDA-MB-231 were cultured in 6-well plates until 80% confluence, then culture medium was changed to control (regular culture medium) or pre-Mn (regular culture medium with the addition of manganese chloride ($MnCl_2$) 5 µM. Cells were incubated in these conditions for 1 h. Next, medium (control or Mn-added) was removed, cells were rinsed and mechanically removed from the plate in a cross-shaped pattern using a sterile P1000 tip. Cells were rinsed again for removal of cell debris and incubated in control culture medium for 12 h. In order to evaluate cell migration and wound closure, cells were imaged, using the cross center as a reference at 0 h and 12 h. Migration was quantified using ImageJ software and expressed as the percentage of migrated distance relative to the original wound width.

X-ray microfluorescence analyses of tumor cell lines HeLa, B16 and MDA-MB-231

X-ray microfluorescence analyses were performed at the Brazilian Synchrotron Light Source Laboratory (LNLS). Analyzed samples comprised of cultured cells placed on ultralene thin film (SPEX SamplePrep, NJ, USA). Cultured cell samples were quantified, scraped from the plate, placed on ultralene film and let air dry. Samples were analyzed at D09B X-ray Fluorescence beamline at room temperature and ambient pressure.

Divalent cations cell toxicity assays

LCC cells were seeded in 24-well plates ($6x10^4$ cells/well) and after cultures reached 80% confluence, cells were incubated for 24 h in increasing concentrations of $MgCl_2$ or $ZnCl_2$ (5, 10, 25, 50, 100 and 500 µM). Cells were harvested by enzymatic treatment and viable cells were quantified in a Neubauer chamber using trypan blue.

Divalent cations wound healing assays

LLC cells were cultured in 6-well plates until 80% confluence, then culture medium was changed to control (regular culture medium) or pre-Mg or pre-Zn (regular

culture medium with the addition of magnesium chloride ($MgCl_2$) or zinc chloride ($ZnCl_2$) at 10 µM and 25 µM. Cells were incubated in these conditions for 1 h. Next, culture medium (control or cation-added) was removed, cells were rinsed and mechanically removed from the plate in a cross-shaped pattern using a sterile P1000 tip. Cells were rinsed again for removal of cell debris and incubated in control culture medium for 12 h. In order to evaluate cell migration and wound closure, cells were imaged, using the cross center as a reference at 0 h and 12 h. Migration was quantified using ImageJ software and expressed as the percentage of migrated distance relative to the original wound width.

Cytometry analyses

For flow cytometry analysis of syndecan-1, LLC cells were prepared according to the cell migration wound healing assay. After 8 h of migration, cells were removed from the plates using PBS/EDTA 1mM solution and fixed with 4% paraformaldehyde. Then, cells were stained with rat anti-mouse syndecan-1 (1:200 – RD systems, MN, USA) followed by anti-mouse IgG Cy5 (1:500 – Life Technologies, CA, USA). Syndecan-1 expression was evaluated in 10,000 events/cells using a FACS LSRFortessa (BD Bioscience, NJ, USA) and analyzed in the Summit software (Cytomation, CO, USA).

Reverse transcription-polymerase chain reaction (RT-PCR) analyses

LLC cells cultured in standard conditions were submitted to a mRNA extraction protocol using TRizol reagent (catalog # 15596026, Invitrogen, CA, USA). Next, the extracted mRNA was treated with DNAse I (catalog # AM2222, Life Technologies, CA, USA) and used for cDNA synthesis using the High Capacity cDNA Reverse Transcription Kit with RNase Inhibitor (catalog # 4374966, Applied Biosystems, CA, USA). All procedures were performed according to manufacturer instructions. The resulting cDNA was amplified using AmpliTaq Gold DNA Polymerase with Buffer II and $MgCl_2$ (catalog # N8080241, Applied Biosystems, CA, USA). Primer sequences and amplicon sizes are as follows: GAPDH forward 5’-CATCTTCCAGGAGCGAGACC, reverse 5’-TGAAGTCGCAGGAGACAACC, amplicon: 609 bp; ITGB1 forward 5’-AGTGCCATGAGGGAAATGGG, reverse 5’- AACAATTCCAGCAACCACGC,

amplicon: 797 bp; SDC1 forward 5’- ATGGCTCTGGGGATGACTCT, reverse 5’-CTCCCAGCACTTCCTTCCTG, amplicon: 663 bp. Amplicons were analyzed using electrophoresis in 1% agarose gel (m/v, TAE buffer, ethidium bromide 25 ng/mL) and 1 Kb Plus DNA Ladder (catalog # 10787-018, Invitrogen, CA, USA) was used as a molecular weight size marker. Electrophoresis was set at 100 V for 30-45 minutes. Next, gels were promptly imaged using an UV gel imaging system (Bio-Rad, CA, USA). Negative controls were as follows: extracted mRNA as template for the RT-PCR (to detect possible genomic DNA contamination) and cDNA-free RT-PCR reactions (to detect possible contaminated reagents).

Heparin-treated animal model of spontaneous metastasis

C57BL/6 mice were inoculated with LLC cells as described for the animal model of spontaneous metastasis, however, at weeks 2 and 4 animals were treated with 100 µL single injections of unfractionated bovine heparin (UFH) 0.1 ng/mL at the tumor site. After 5 weeks of tumor development, tissues were collected for further analyses. All procedures involving animal experimentation were approved by the Federal University of Rio de Janeiro Animal Experimentation Committee (protocol number: 015/18) and were performed in accordance with the Brazilian guidelines for scientific use of animals.

Multi-elemental inductively coupled plasma (ICP)-optical emission spectroscopy

Modified fetal bovine sera (Mn-low and Mn-high FBS) were submitted to multi-elemental ICP-OES analyses for the investigation of actual elemental concentrations post modification protocol. Analyses were performed using an Optima DV 4300 (Perkin Elmer, Waltham, MA, USA) ICP-OES. The following parameters were used for the analyses: peristaltic pump flow 1.5 mL/min, plasma flow 15 L/min, nebulization flow 0.6 L/min, 1400 W, radial plasma view for Ca and K, axial plasma view for Cu, Fe, Mn, P and Zn. Multi-elemental calibration curve ranged from 0.001 to 20.0 mg/L.

Supplementary Figures

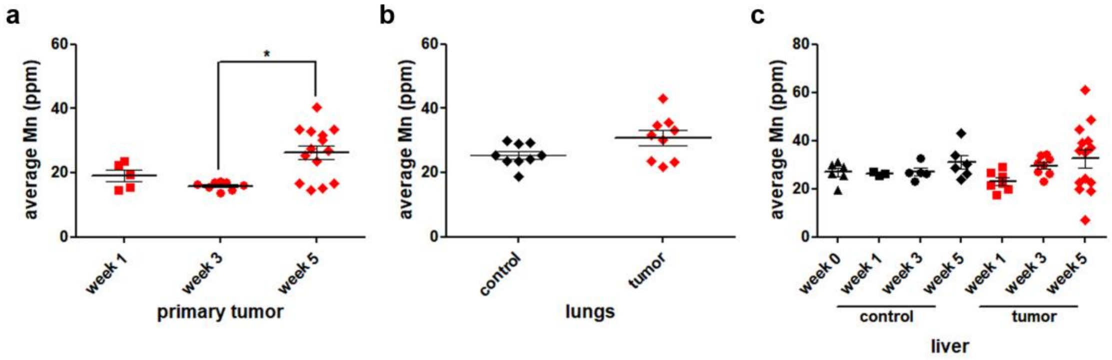

Fig. S1. Primary tumors present elevated levels of average manganese, while distant organs remain unchanged. Average Mn mass fraction from (a) primary tumors, (b) lungs and (c) livers were plotted. Units are expressed in mass fraction as ppm (parts per million). *$p<0.05$ Kruskal-Wallis test and Dunn's multiple comparison post-test. Primary tumors (week 1) N=5, (week 3) N=9, (week 5) N=17; Lungs (control) N=9, (tumor-bearing) N=10; Livers (control week 0) N=6, (control week 1) N=3, (tumor-bearing week 1) N=7, (control week 3) N=5, (tumor-bearing week 3) N=8, (control week 5) N=6, (tumor-bearing week 5) N=15.

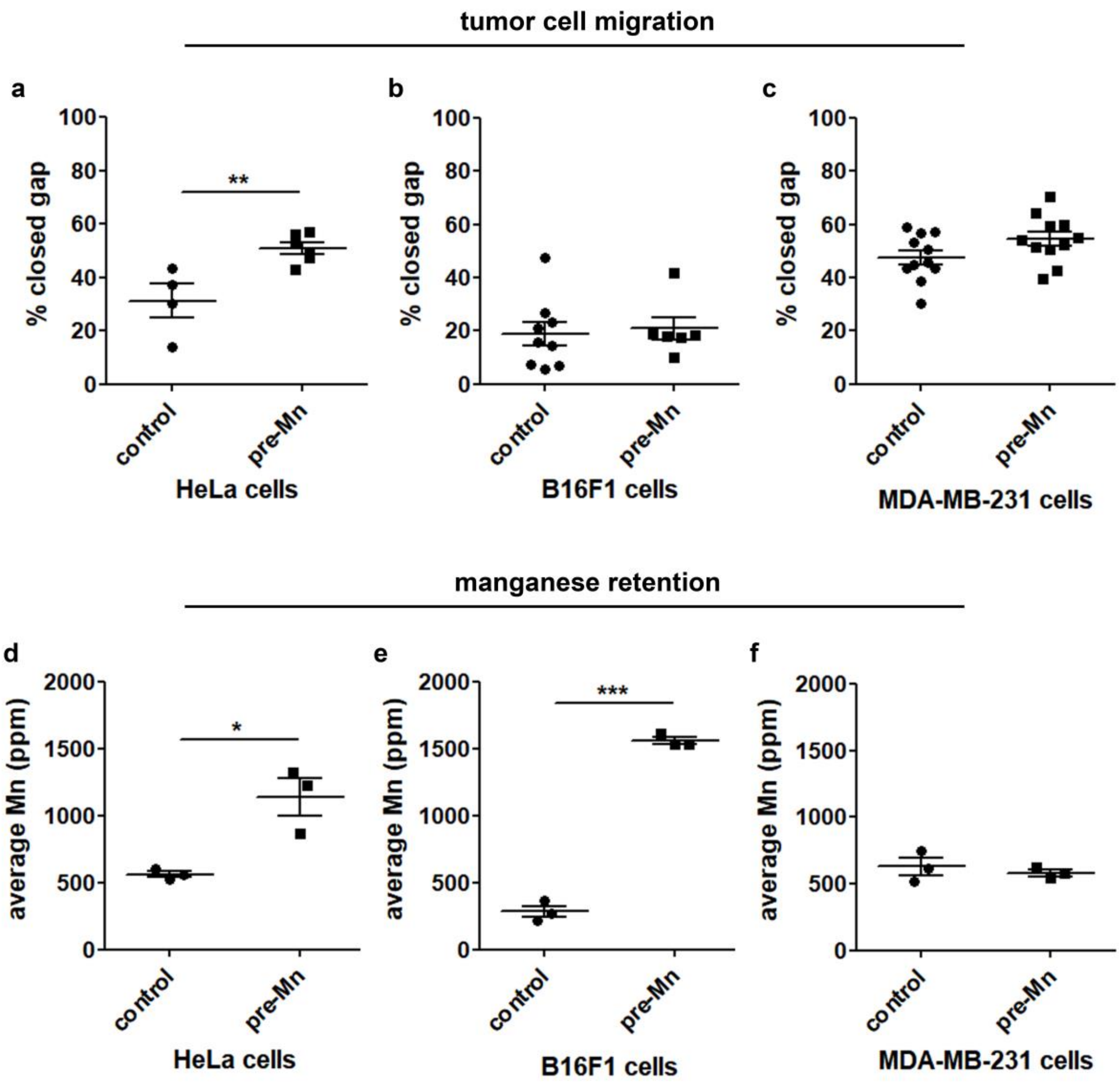

Fig. S2. Manganese retention and its effects on cell migration of other tumor cell lines. Different tumor cell lines were evaluated for migration pattern using wound healing assays by (a-c) intermittent monitoring. The tested cell lines were (a) HeLa, (b) B16F1 and (c) MDA-MB-231. Mn content was evaluated by briefly treating cells with $MnCl_2$ 5 μM for 1 h and collecting cells for XRF analyses after 3 h of incubation in standard medium. The analyzed cell lines were (d) HeLa, (e) B16F1 and (f) MDA-MB-231. Wound healing assays HeLa control N=4, HeLa pre-Mn N=6; B16F1 control N=9, pre-Mn N=6; MDA-MB-231 control and pre-Mn N=3. *p<0.05, **p<0.01, ***p<0.0001; Student's unpaired t-test.

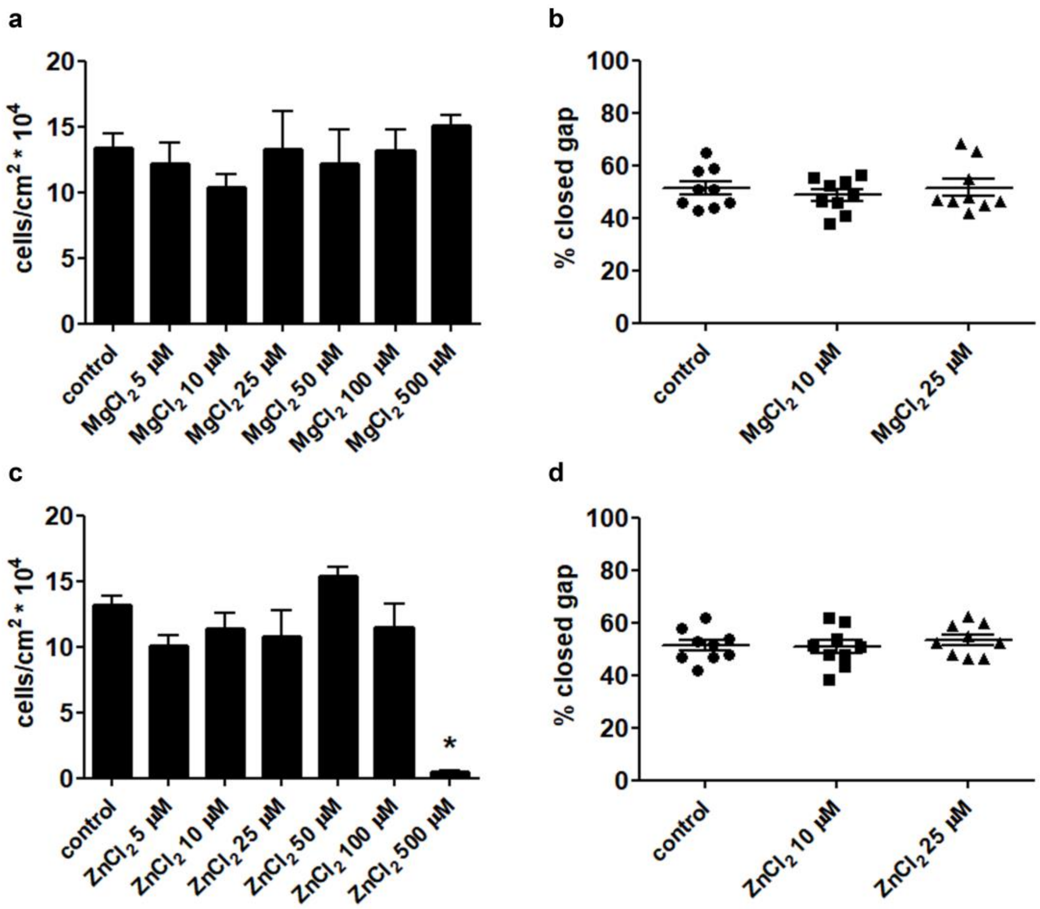

Fig. S3. Magnesium and zinc do not affect tumor cell growth, survival and migration at low concentrations. LLC cell growth and survival were evaluated after 24 h of incubation with different (a) $MgCl_2$ and (c) $ZnCl_2$ concentrations. Cell number represents adhered live cells only. LLC cell migration was evaluated in wound healing assays by pre-incubating cells in (b) $MgCl_2$ or (c) $ZnCl_2$ at 10 µM and 25 µM for 1 h, followed by rinsing and incubation in control medium. Cells were imaged at 0 h and 12 h of migration. Cell growth assay N=3, wound healing assay N=9. *$p<0.05$, one-way ANOVA and Bonferroni's multiple comparison post-test.

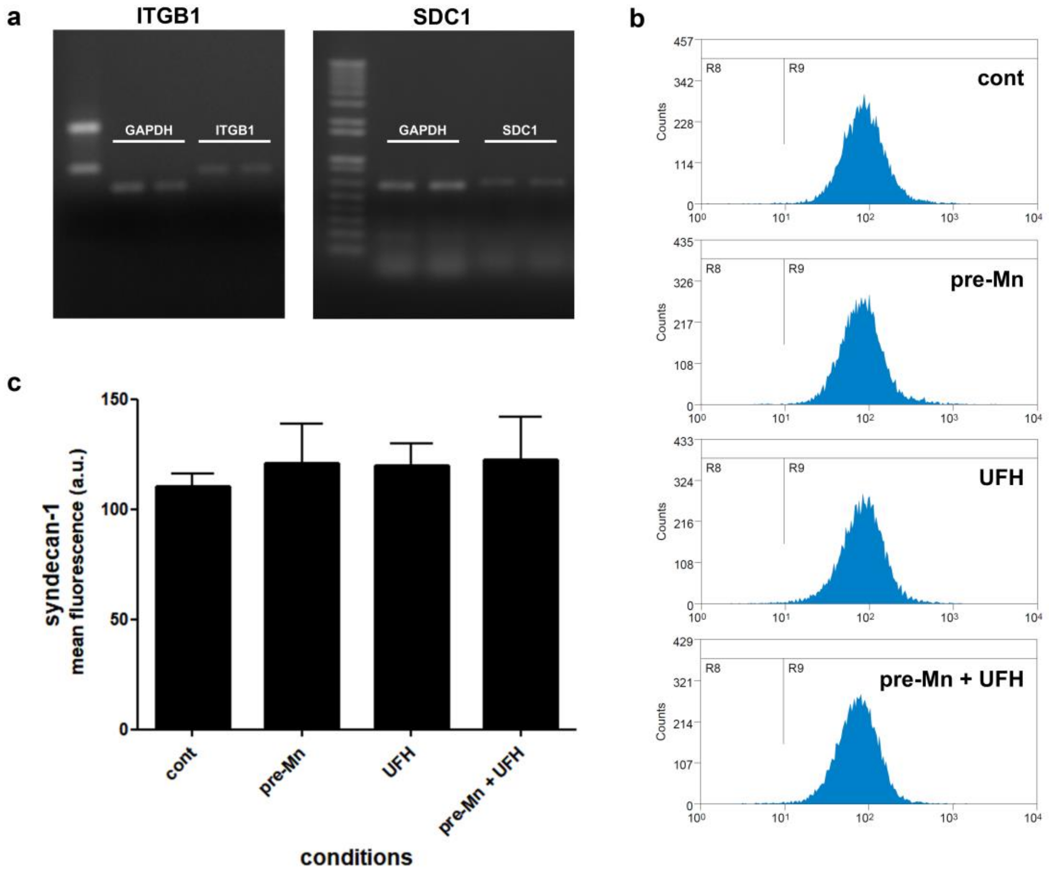

Fig. S4. Detection of β1-integrin and syndecan-1 in LLC cells. (a) PCR analyses were performed in LLC cells cultured in standard conditions to evaluate the expression of ITGB1 and SDC1. Molecular-weight size marker 1 kb. Next, LLC cells were submitted to the same conditions as a wound healing assay and collected at 8 h of migration for (b) flow cytometry analyses. (c) Mean fluorescence intensity. UFH (bovine unfractionated heparin – 0.1 ng/mL); pre-Mn ($MnCl_2$ 5 µM 1 h pretreatment prior to migration). PCR N=2; cytometry N=3. Statistical analyses: one-way ANOVA test and Bonferroni's multiple comparison post-test.

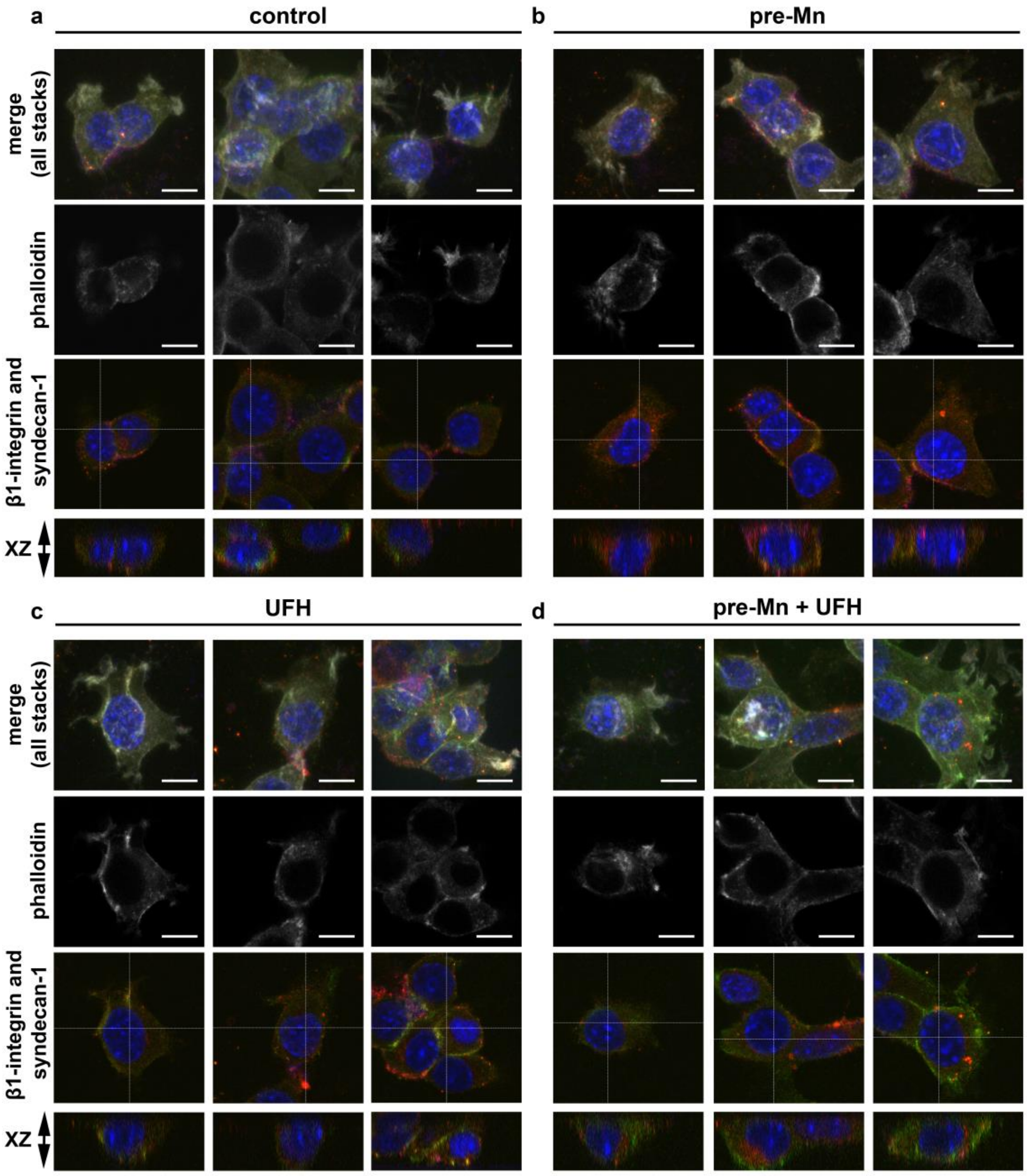

Fig. S5. Manganese affects β1-integrin and syndecan-1 expression in tumor cells. Confocal microscopy images of immunostainings for β1-integrin, syndecan-1 and F-actin (phalloidin). DAPI (blue), β1-integrin (red), syndecan-1 (green) and phalloidin (white). (a) control; (b) pre-Mn ($MnCl_2$ 5 μM 1h pretreatment prior to migration); (c) UFH (bovine unfractionated heparin – 0.1 ng/mL); (d) pre-Mn + UFH. XZ axis projections were generated from higher magnification images of β1-integrin and syndecan-1 merged stainings. Scale bars are 10 μm. N=6.

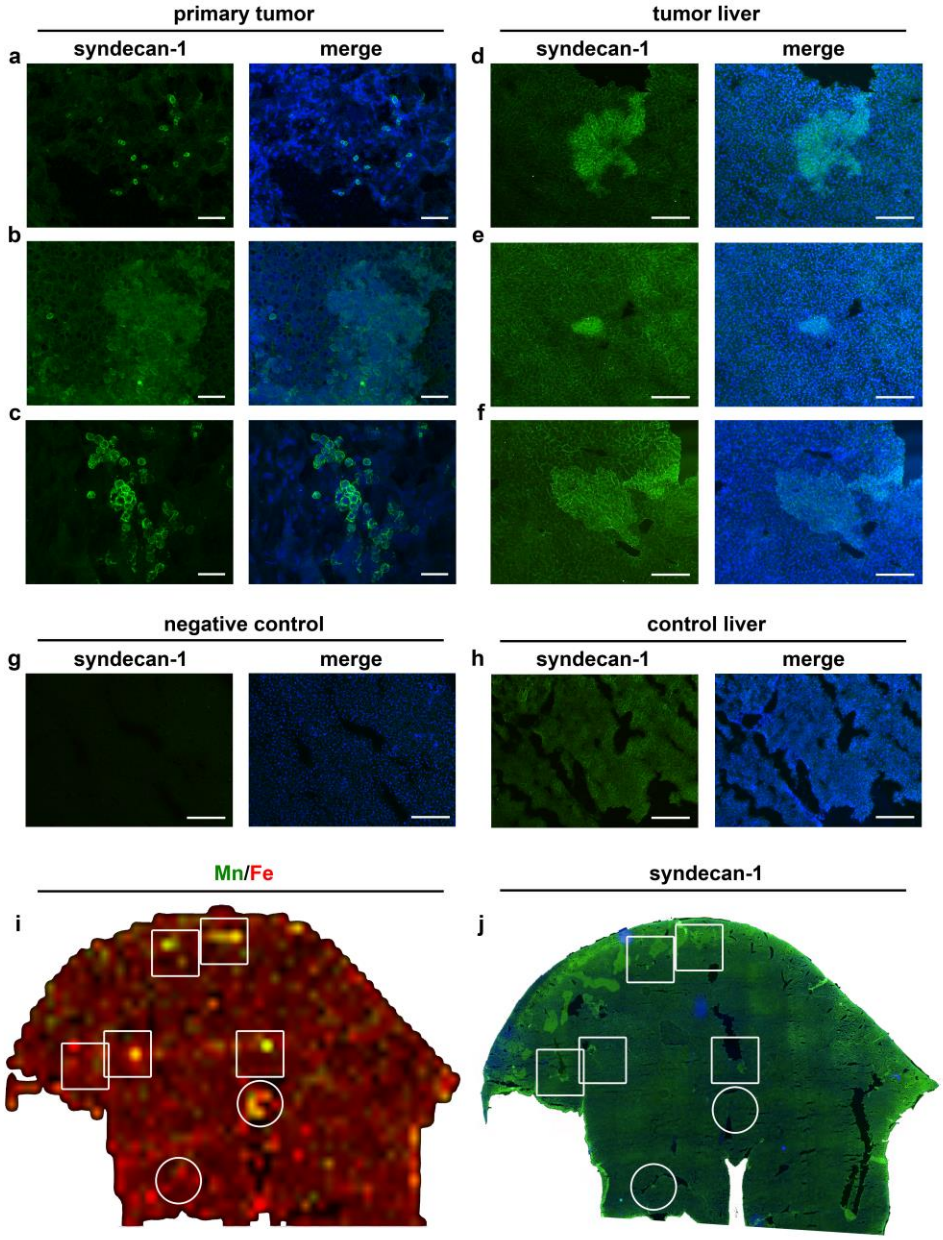

Fig. S6. Syndecan-1 is highly expressed in small cell clusters in primary tumors and tumor-bearing mice livers. Representative images of syndecan-1 immunostainings from (a, b and c) primary tumors, (d, e and f) tumor-bearing mice livers and (h) control liver, all at week 5. (g) Negative control. (i) Mn – green – and Fe – red – distribution map of a tumor-bearing mouse liver. (j) Syndecan-1 immunostaining of sequential liver section shown in (i). Scale bars are 25 µm (a, b, c) and 50 µm (d to h). Primary tumor N=4; Tumor-bearing mice livers N=6; Control mice livers N=4.

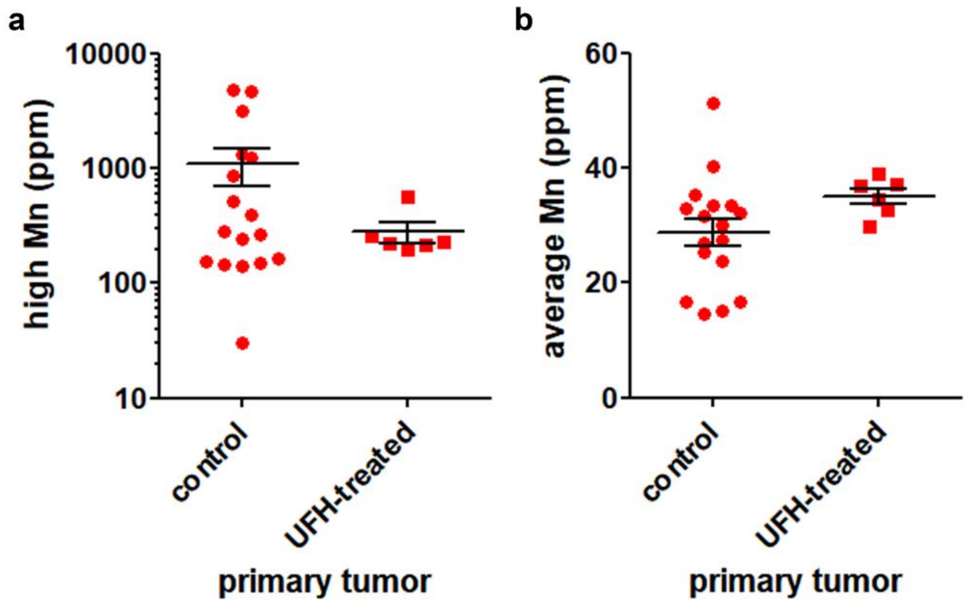

Fig. S7. Manganese content in primary tumors treated with unfractionated heparin. Tumor-bearing mice received unfractionated heparin (UFH) injections at weeks 2 and 4 of tumor progression and primary tumors were collected at week 5 of tumor progression for XRF analyses. Control animals N=17, UFH-treated N=6. Student's unpaired t-test non-significant.

Table S1. Average manganese mass fraction in tissue sections from C57BL/6 mice.

	**Mn (ppm) ± SEM**	
**organs**	**control mice**	**tumor-bearing mice**
**primary tumor – week 1**	NA	19 ± 2
**primary tumor – week 3**	NA	15.9 ± 0.4
**primary tumor – week 5**	NA	26 ± 2
**lungs – week 5**	26 ± 2	29 ± 3
**liver – week 0**	27 ±2	NA
**liver – week 1**	26.3 ± 0.4	23 ± 2
**liver – week 3**	27 ±2	30 ± 1
**liver – week 5**	31 ±3	31 ± 3
**peripheral blood – week 0**	$(40 \pm 6)10^2$	NA
**peripheral blood – week 1**	$(46 \pm 4)10^2$	$(49 \pm 3)10^2$
**peripheral blood – week 3**	$(40 \pm 5)10^2$	$(43 \pm 4)10^2$
**peripheral blood – week 5**	$(43 \pm 4)10^2$	$(21 \pm 1)10^2$
**bone marrow – week 3**	(21 ± 6)10	(16 ± 4)10
**bone marrow – week 5**	(21 ± 6)10	(35 ± 6)10

*NA, non-applicable

Table S2. High manganese regions in tissue sections from C57BL/6 mice.

	Mn (ppm) ± SEM	
**organs**	**control mice**	**tumor-bearing mice**
**primary tumor – week 1**	NA	(16 ± 4)10
**primary tumor – week 3**	NA	(5 ± 2) 10^2
**primary tumor – week 5**	NA	(11 ± 4) 10^2
**lungs – week 5**	113 ± 4	(7 ± 2) 10^2
**liver – week 0**	(31 ± 4)10	NA
**liver – week 1**	(49 ± 8)10	(7 ± 3) 10^2
**liver – week 3**	(15 ± 4) 10^2	(5± 4) 10^3
**liver – week 5**	(5 ± 2) 10^2	(13 ± 5) 10^2

*NA, non-applicable

Table S3. Multi-elemental ICP-OES analyses of modified fetal bovine sera

Elements	Reference values	Control FBS	Mn-high FBS	Mn-low FBS
P	2–5 mM*;<br>4 mM***	3.40 ± 0.03 mM	3.40 ± 0.03 mM	3.50 ± 0.03 mM
K	5–15 mM*;<br>5.4 mM***	12.0 ± 0.1 mM	16.7 ± 0.2 mM	17.3 ± 0.1 mM
Ca	4–7 mM*;<br>1.4 mM***	3.50 ± 0.03 mM	3.30 ± 0.03 mM	3.30 ± 0.04 mM
Zn	0.1–1.0 µM*;<br>42 µM***	36.1 ± 0.4 µM	35.0 ± 0.3 µM	34.9 ± 0.3 µM
Mn	0.5–1.2 µM**	0.80 ± 0.02 µM	7.70 ± 0.06 µM	0.60 ± 0.01 µM
Fe	10–50 µM*	46.3 ± 0.5 µM	45.4 ± 0.4 µM	45.1 ± 0.5 µM
Cu	2.5 µM***	2.40 ± 0.03 µM	2.90 ± 0.02 µM	2.80 ± 0.03 µM

Values extracted from *[1], **[2] and ***[3]

1. Defined Media and Supplements. in *Culture of Animal Cells* 99–114 (John Wiley & Sons, Ltd, 2010). doi:10.1002/9780470649367.ch8.
2. Vicogne, D. *et al.* Fetal Bovine Serum impacts the observed N-glycosylation defects in TMEM165 KO HEK cells. *J Inherit Metab Dis* **43**, 357–366 (2020).
3. Glassman, A. B., Rydzewski, R. S. & Bennett, C. E. Trace metal levels in commercially prepared tissue culture media. *Tissue and Cell* **12**, 613–617 (1980).

Table S4. Glycosaminoglycans bind to manganese

samples	Mn (ppm) ± SEM
control water	3.3 ± 0.3
unfractionated heparin (UFH)	92 ± 4
ascidian DS	88 ± 12

Movie S1. Time lapse imaging of Lewis lung carcinoma (LLC) cells migration in control conditions.

Movie S2. Time lapse imaging of Lewis lung carcinoma (LLC) cells migration after brief exposure to $MnCl_2$.

Movie S3. Time lapse imaging of Lewis lung carcinoma (LLC) cells migration with unfractionated heparin incubation.

Movie S4. Time lapse imaging of Lewis lung carcinoma (LLC) cells migration after brief exposure to $MnCl_2$ followed by incubation with unfractionated heparin incubation during migration.
